# Supplementary material for: A network medicine approach to investigation and population-based validation of disease manifestations and drug repurposing for COVID-19
Source: PLoS Biol. 2020 Nov 6;18(11):e3000970. doi: 10.1371/journal.pbio.3000970 (PMC7728249; doi:10.1371/journal.pbio.3000970)
Supplement: S1 Fig — In total, 246 differentially expressed genes (DEGs) in human bronchial epithelial cells infected with SARS-CoV-2 were obtained from the NCBI GEO database with the accession number GSE147507, denoted as SARS2-DEG. The data underlying this figure can be found in S7 Data. (PDF) [file pbio.3000970.s012.pdf]

S1 Fig

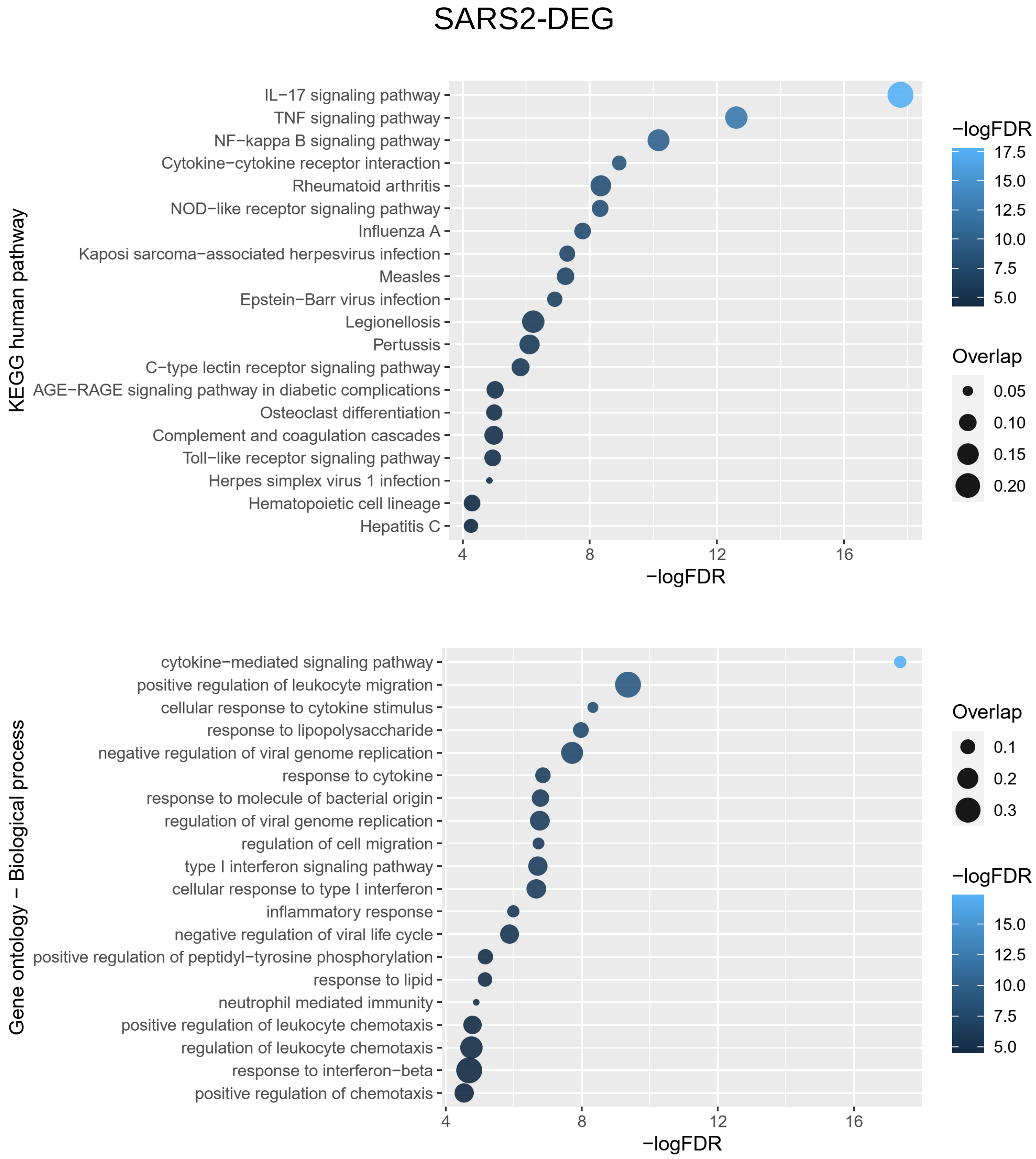

S1 Fig. Functional enrichment analysis for SARS2-DEG. In total, 246 differentially expressed genes (DEGs) in human bronchial epithelial cells infected with SARS-CoV-2 were obtained from the NCBI GEO database with the accession number GSE147507, denoted as SARS2-DEG. The data underlying this figure can be found in S7 Data.
