# Supplementary material for: A network medicine approach to investigation and population-based validation of disease manifestations and drug repurposing for COVID-19
Source: PLoS Biol. 2020 Nov 6;18(11):e3000970. doi: 10.1371/journal.pbio.3000970 (PMC7728249; doi:10.1371/journal.pbio.3000970)
Supplement: S2 Fig — In total, 293 differentially expressed proteins (DEPs) in human Caco-2 cells infected with SARS-CoV-2 were obtained from Bojkova et al. [22], denoted as SARS2-DEP. The data underlying this figure can be found in S7 Data. (PDF) [file pbio.3000970.s013.pdf]

# S2 Fig

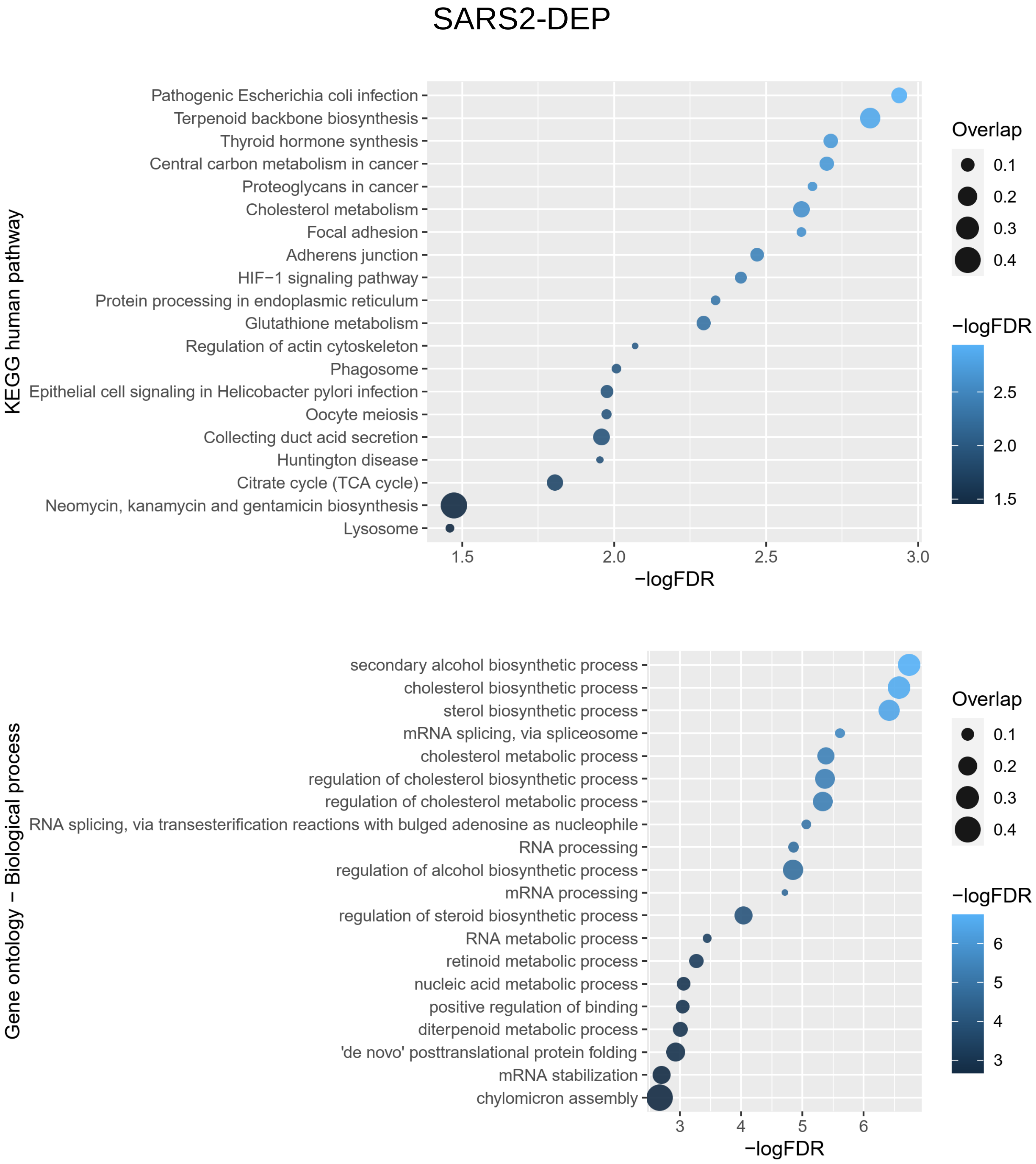


**S2 Fig. Functional enrichment analysis for SARS2-DEP.** In total, 293 differentially expressed proteins (DEPs) in human Caco-2 cells infected with SARS-CoV-2 were obtained from Bojkova, D. *et al.* [22], denoted as SARS2-DEP. The data underlying this figure can be found in S7 Data.
