# Supplementary material for: A network medicine approach to investigation and population-based validation of disease manifestations and drug repurposing for COVID-19
Source: PLoS Biol. 2020 Nov 6;18(11):e3000970. doi: 10.1371/journal.pbio.3000970 (PMC7728249; doi:10.1371/journal.pbio.3000970)
Supplement: S3 Fig — This dataset contains 134 strong literature-evidence-based pan-human coronavirus target host proteins from Zhou et al. [30] with 15 newly curated proteins, denoted as HCoV-PPI. The data underlying this figure can be found in S7 Data. (PDF) [file pbio.3000970.s014.pdf]

S3 Fig

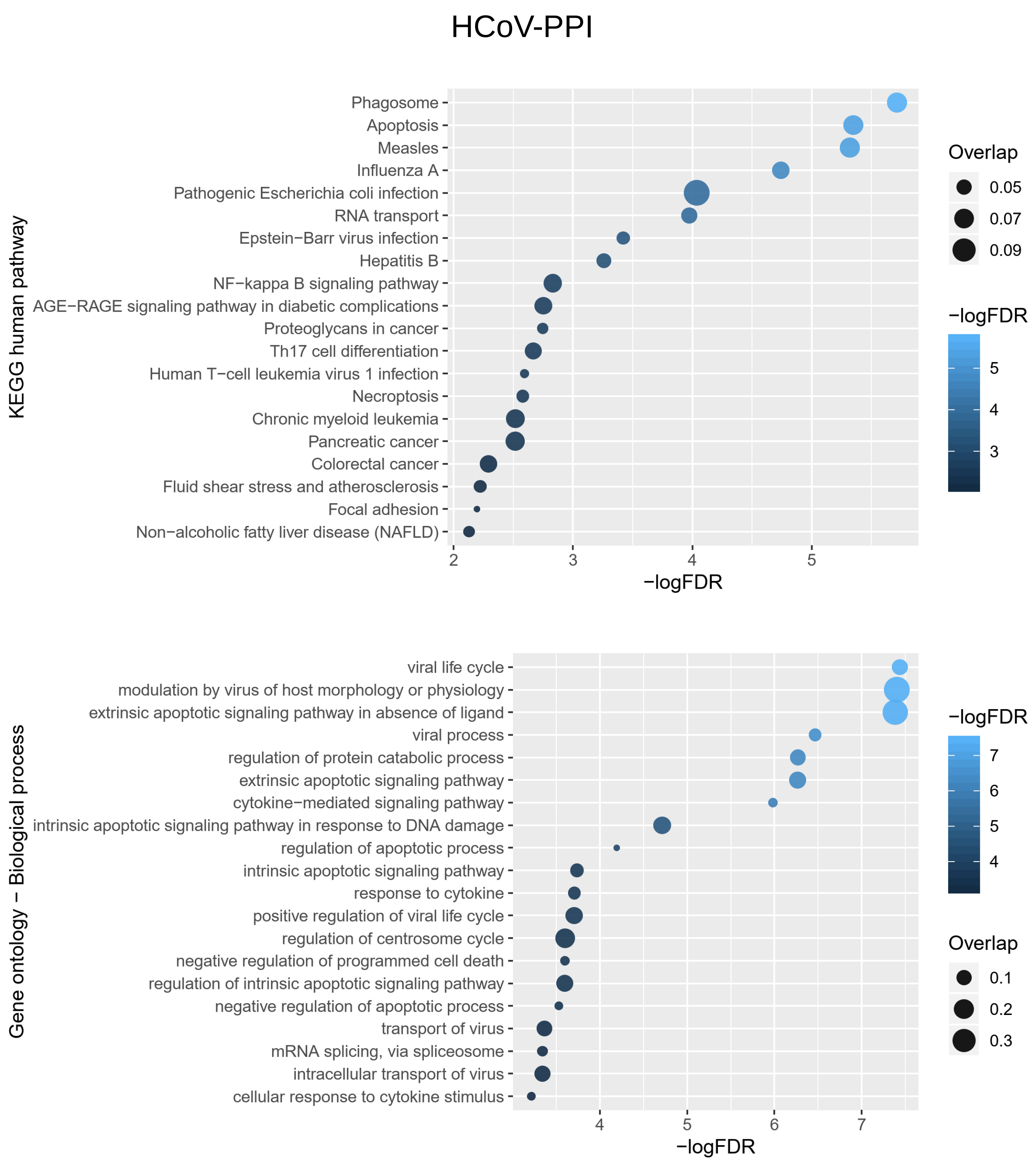

S3 Fig. Functional enrichment analysis for HCoV-PPI. This dataset contains 134 strong literature-evidence-based pan-human coronavirus target host proteins from Zhou, Y. *et al.* [30] with 15 newly curated proteins, denoted as HCoV-PPI. The data underlying this figure can be found in S7 Data.
