# Supplementary material for: A network medicine approach to investigation and population-based validation of disease manifestations and drug repurposing for COVID-19
Source: PLoS Biol. 2020 Nov 6;18(11):e3000970. doi: 10.1371/journal.pbio.3000970 (PMC7728249; doi:10.1371/journal.pbio.3000970)
Supplement: S4 Fig — This dataset contains 332 proteins involved in protein–protein interactions with 26 SARS-CoV-2 viral proteins identified by affinity purification–mass spectrometry from Gordon et al. [8], denoted as SARS2-PPI. The data underlying this figure can be found in S7 Data. (PDF) [file pbio.3000970.s015.pdf]

S4 Fig

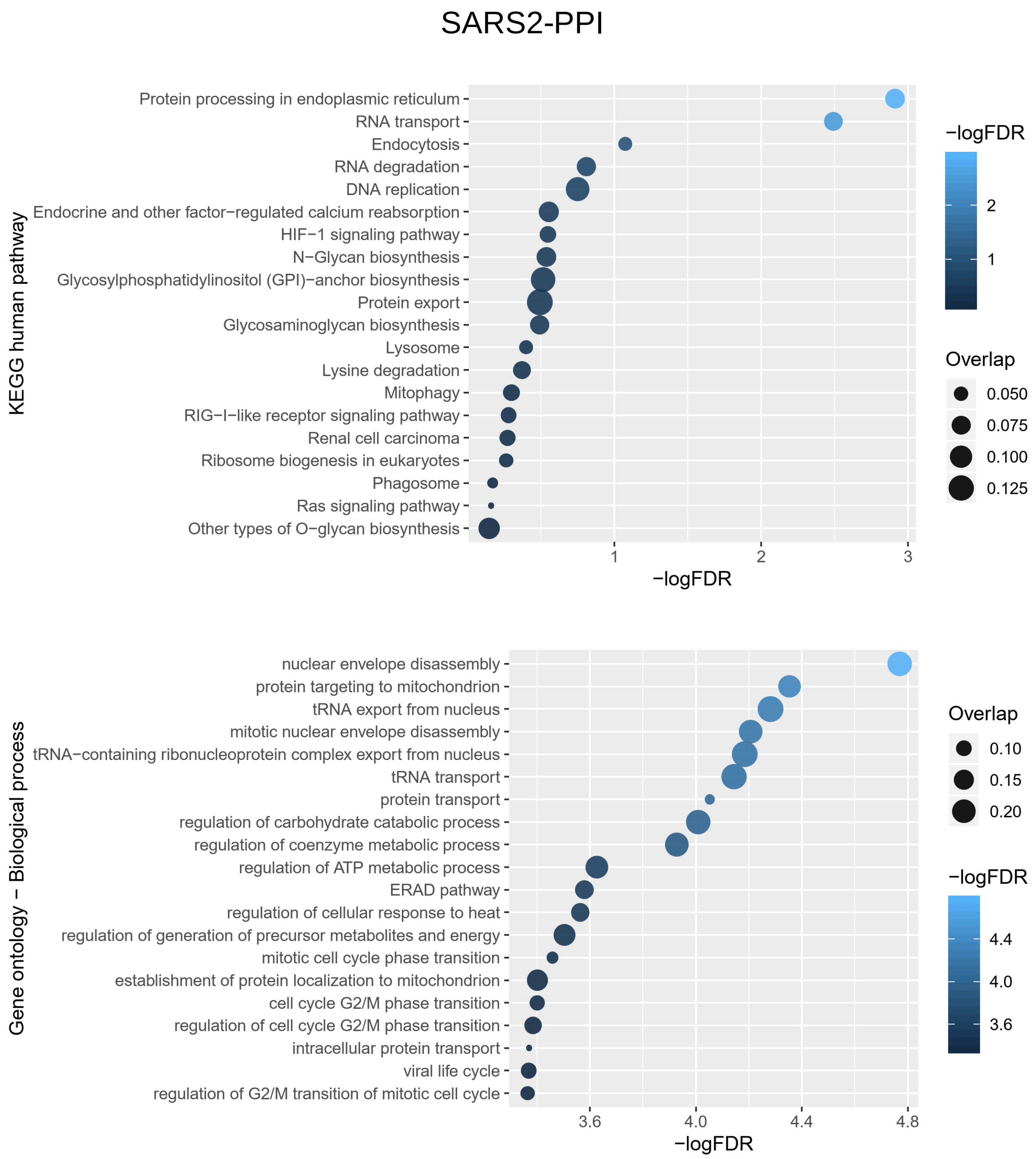

S4 Fig. Functional enrichment analysis for SARS2-PPI. This dataset contains 332 proteins involved in protein-protein interactions with 26 SARS-CoV-2 viral proteins identified by affinity purification-mass spectrometry from Gordon, D. E. *et al.* [8], denoted as SARS2-PPI. The data underlying this figure can be found in S7 Data.
