# Supplementary material for: A network medicine approach to investigation and population-based validation of disease manifestations and drug repurposing for COVID-19
Source: PLoS Biol. 2020 Nov 6;18(11):e3000970. doi: 10.1371/journal.pbio.3000970 (PMC7728249; doi:10.1371/journal.pbio.3000970)
Supplement: S5 Fig — Due to the interactome nature of HCoV-PPI and SARS2-PPI, we combined these datasets as the fifth SARS-CoV-2 dataset, which has 460 proteins and is denoted as PanCoV-PPI. The data underlying this figure can be found in S7 Data. (PDF) [file pbio.3000970.s016.pdf]

S5 Fig

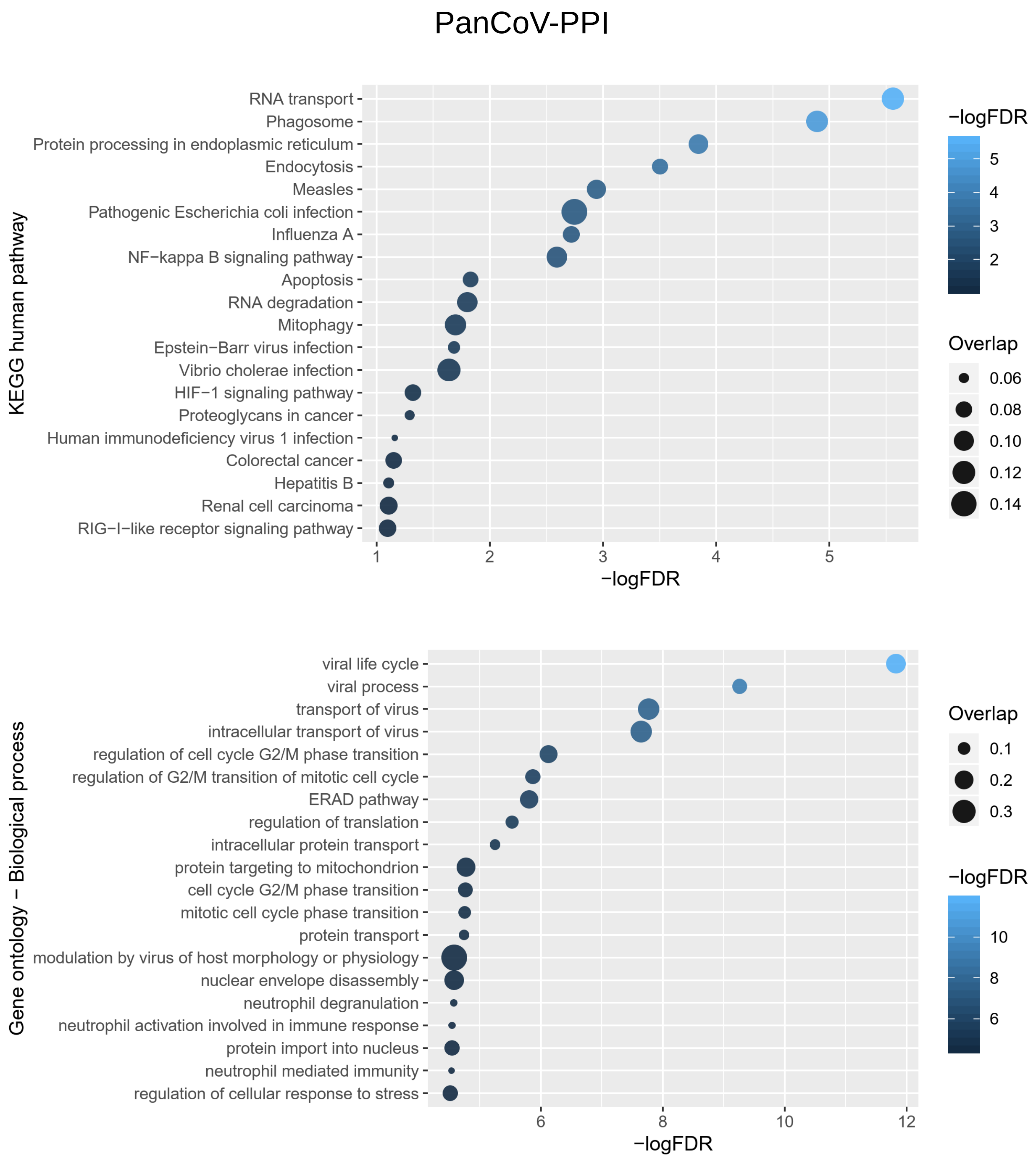

S5 Fig. Functional enrichment analysis for PanCoV-PPI. Due to the interactome nature of HCoV-PPI and SARS2-PPI, we combined these datasets as the fifth SARS-CoV-2 dataset, which has 460 proteins and is denoted as PanCoV-PPI. The data underlying this figure can be found in S7 Data.
