# Supplementary material for: A network medicine approach to investigation and population-based validation of disease manifestations and drug repurposing for COVID-19
Source: PLoS Biol. 2020 Nov 6;18(11):e3000970. doi: 10.1371/journal.pbio.3000970 (PMC7728249; doi:10.1371/journal.pbio.3000970)
Supplement: S6 Fig — Node degree (blue), dN/dS ratio (orange), evolutionary ratio (green), and lung expression specificity (purple) are shown for each dataset. Grey areas indicate mean ± standard deviation of 100 repeats using randomly selected genes. The data underlying this figure can be found in S8 Data. (PDF) [file pbio.3000970.s017.pdf]

S6 Fig

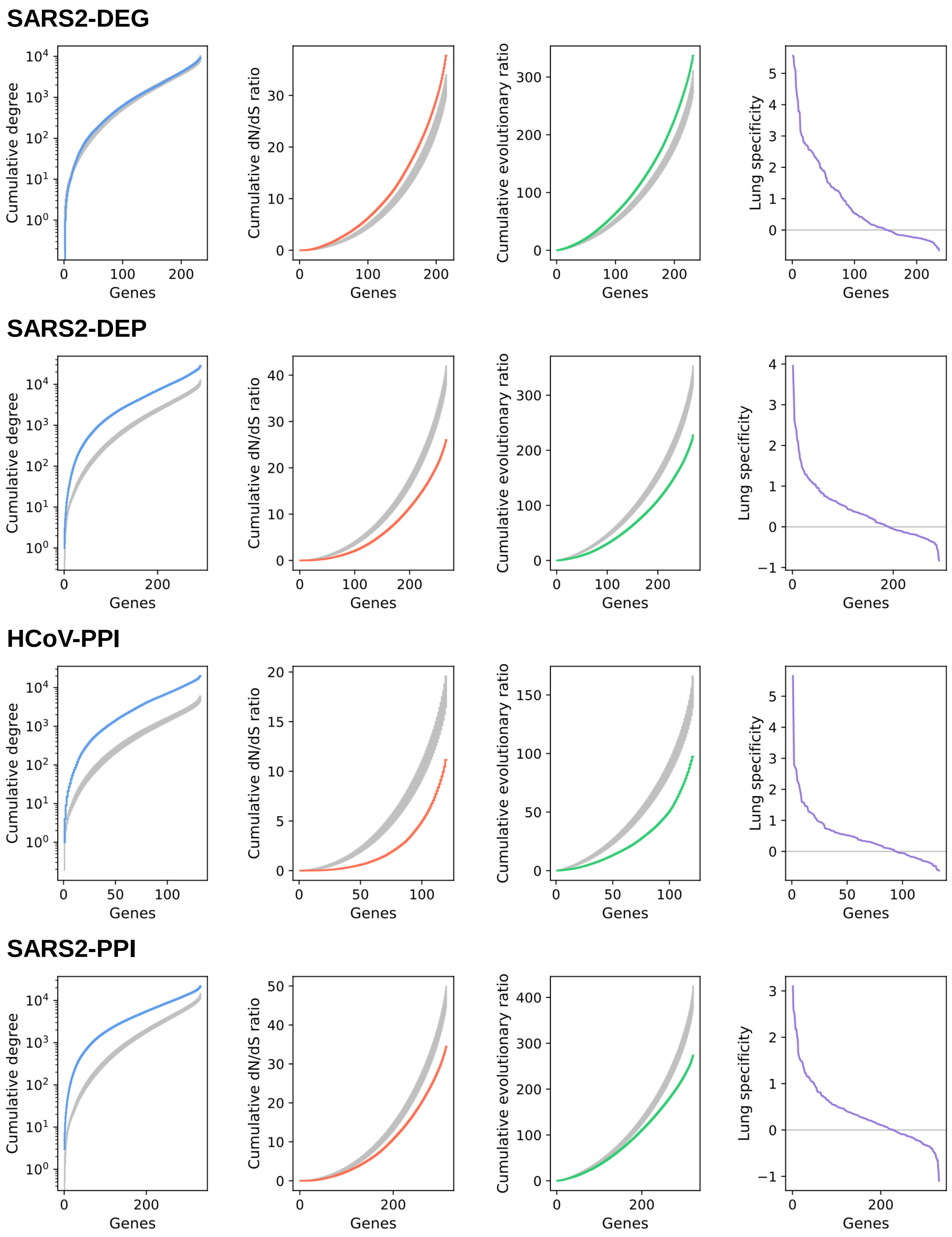

S6 Fig. Characteristics of the four SARS-CoV-2 target datasets. Node degree (blue), *dN/dS* ratio (orange), evolutionary ratio (green), and lung expression specificity (purple) are shown for each dataset. Grey areas indicate mean ± standard deviation of 100 repeats using randomly selected genes. The data underlying this figure can be found in S8 Data.
