# Supplementary material for: A network medicine approach to investigation and population-based validation of disease manifestations and drug repurposing for COVID-19
Source: PLoS Biol. 2020 Nov 6;18(11):e3000970. doi: 10.1371/journal.pbio.3000970 (PMC7728249; doi:10.1371/journal.pbio.3000970)
Supplement: S7 Fig — (A) The risk of chronic obstructive pulmonary disease (COPD) is increased in severe COVID-19 patients. (B) Subnetwork shows the proteins potentially involved in the interaction between COPD and COVID-19. The data underlying this figure can be found in S9 Data. (PDF) [file pbio.3000970.s018.pdf]

## S7 Fig

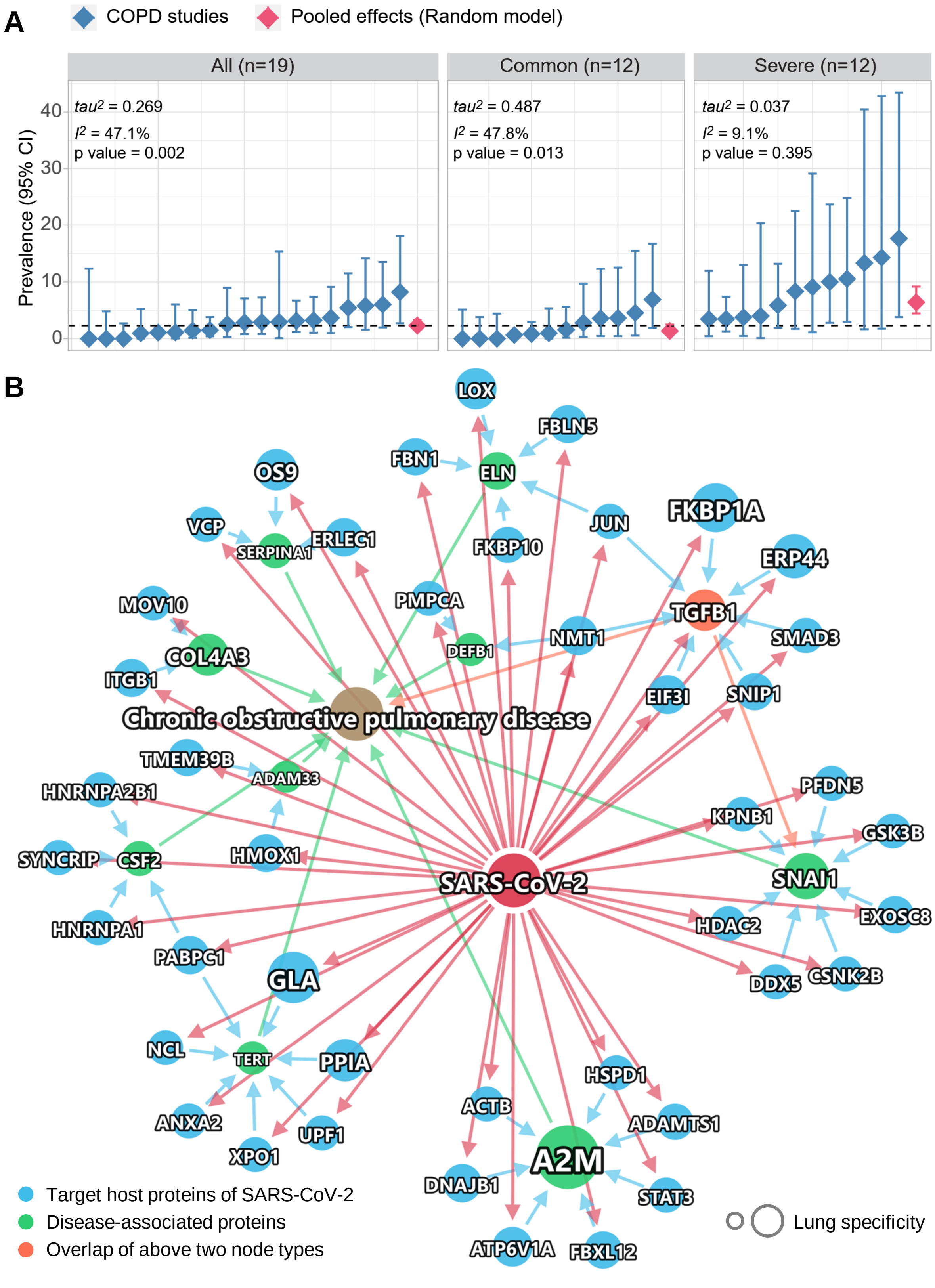


**S7 Fig. Chronic obstructive pulmonary disease and COVID-19.** (**A**) The risk of chronic obstructive pulmonary disease (COPD) is increased in severe COVID-19 patients. (**B**) Subnetwork shows the proteins potentially involved in the interaction between COPD and COVID-19. The data underlying this figure can be found in S9 Data.
