# Supplementary material for: A network medicine approach to investigation and population-based validation of disease manifestations and drug repurposing for COVID-19
Source: PLoS Biol. 2020 Nov 6;18(11):e3000970. doi: 10.1371/journal.pbio.3000970 (PMC7728249; doi:10.1371/journal.pbio.3000970)
Supplement: S8 Fig — (A) The risk of dyspnea is increased in patients with severe COVID-19. (B) UMAP visualization for human bronchial epithelial cells. (C and D) Expression levels of ACE2 across 14 cell types. (E and F) Expression levels of TMPRSS2 across 14 cell types. (G) UMAP visualization for lung cells. (H and I) Expression levels of ACE2 across 9 cell types. (J and K) Expression levels of TMPRSS2 across 9 cell types. The single-cell data with cell type annotation were retrieved from Lukassen et al. [14], which contains 39,778 lung cells and 17,451 bronchial epithelial cells. (PDF) [file pbio.3000970.s019.pdf]

S8 Fig

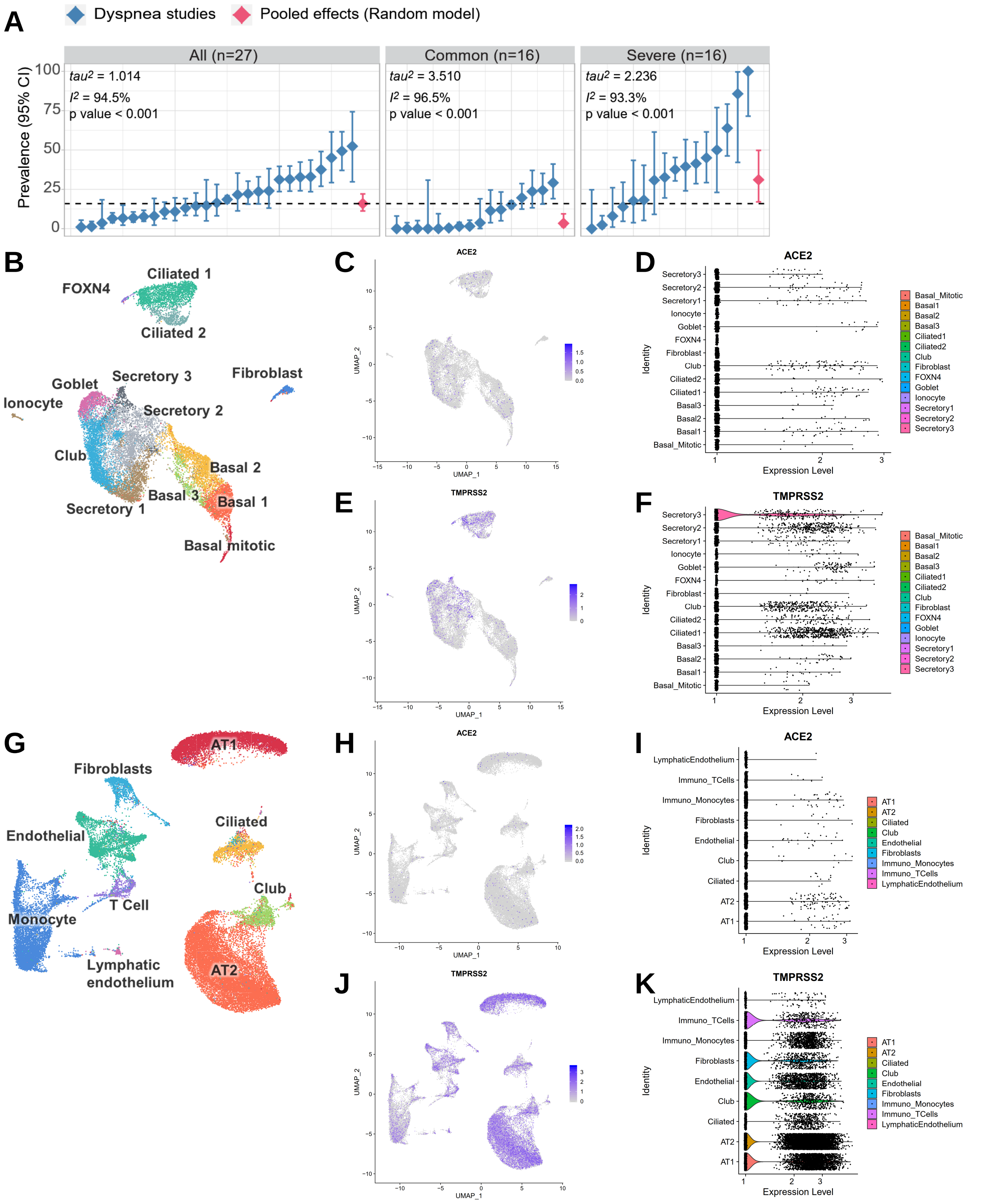

S8 Fig. Asthma and COVID-19. (**A**) The risk of dyspnea is increased in patients with severe COVID-19. (**B**) UMAP visualization for human bronchial epithelial cells. (**C, D**) Expression levels of *ACE2* across 14 cell types. (**E, F**) Expression levels of *TMPRSS2* across 14 cell types. (**G**) UMAP visualization for lung cells. (**H, I**) Expression levels of *ACE2* across 9 cell types. (**J, K**) Expression levels of *TMPRSS2* across 9 cell types. The single cell data with cell type annotation were retrieved from Lukassen, S. *et al.* [14], which contains 39,778 lung cells and 17,451 bronchial epithelial cells.
