# Supplementary material for: A network medicine approach to investigation and population-based validation of disease manifestations and drug repurposing for COVID-19
Source: PLoS Biol. 2020 Nov 6;18(11):e3000970. doi: 10.1371/journal.pbio.3000970 (PMC7728249; doi:10.1371/journal.pbio.3000970)
Supplement: S9 Fig — The expression levels of the genes from the asthma–COVID-19 subnetwork in bronchial epithelial cells (A) and lung cells (B) are shown. (PDF) [file pbio.3000970.s020.pdf]

S9 Fig

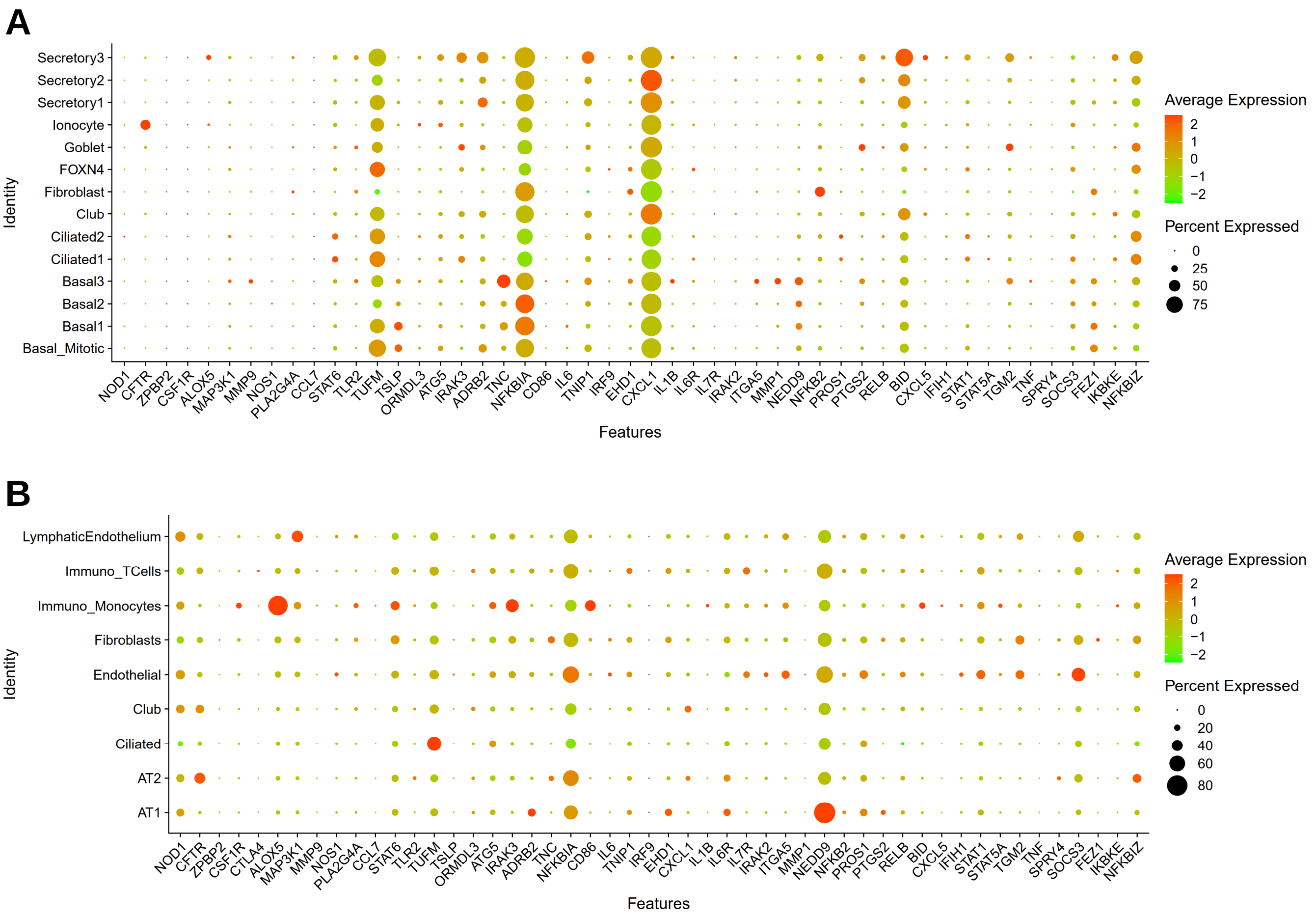

S9 Fig. The expression of asthma genes and SARS-CoV-2 targets. The expression levels of the genes from the asthma-COVID-19 subnetwork in bronchial epithelial cells (**A**) and lung cells (**B**) are shown.
