# Supplementary material for: A network medicine approach to investigation and population-based validation of disease manifestations and drug repurposing for COVID-19
Source: PLoS Biol. 2020 Nov 6;18(11):e3000970. doi: 10.1371/journal.pbio.3000970 (PMC7728249; doi:10.1371/journal.pbio.3000970)
Supplement: S10 Fig — Abdominal pain (A) and diarrhea (B) have increased risks in patients with severe COVID-19. (PDF) [file pbio.3000970.s021.pdf]

S10 Fig

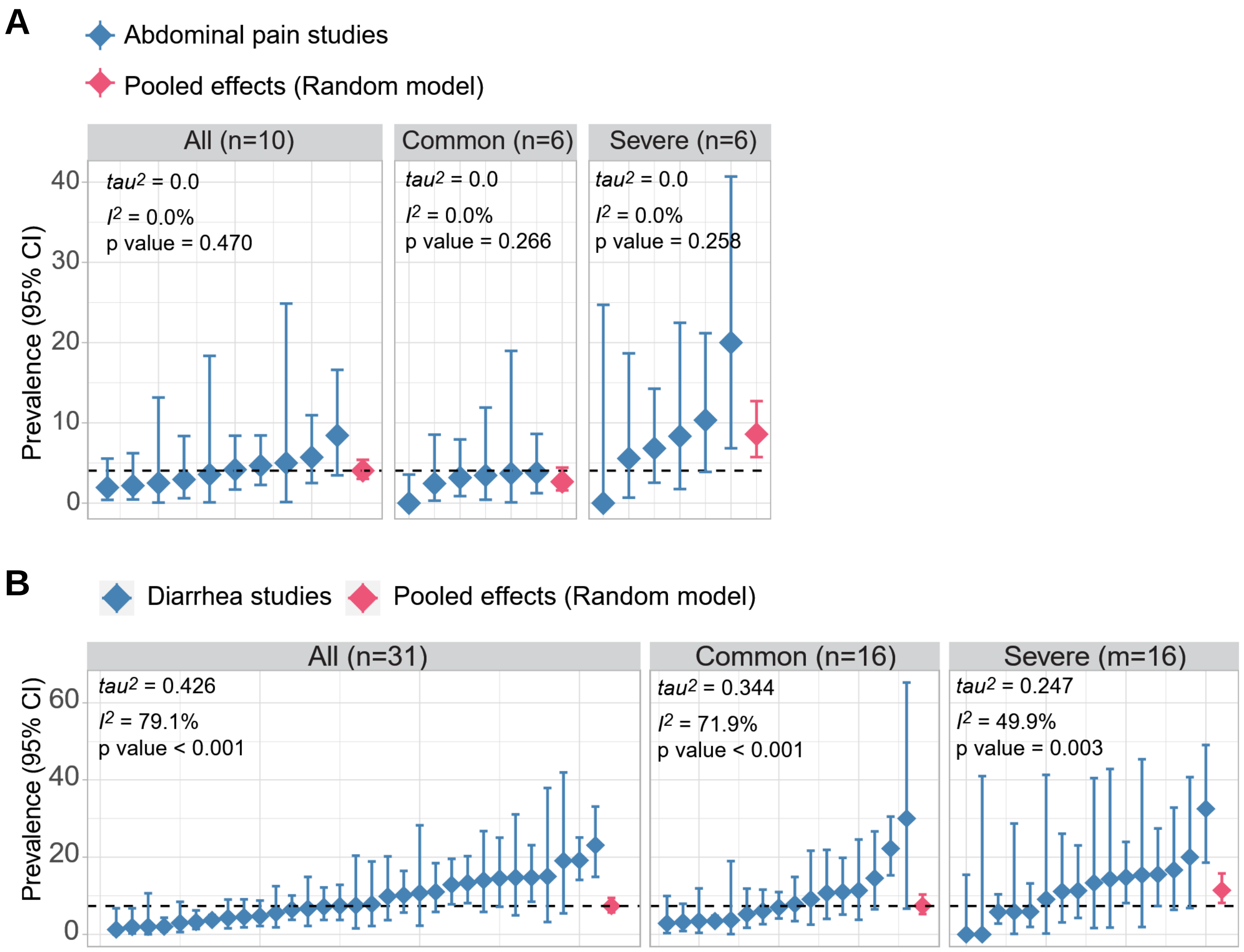

S10 Fig. Risk ratios for abdominal pain and diarrhea in COVID-19 patients. Abdominal pain (**A**) and diarrhea (**B**) have increased risks in patients with severe COVID-19.
