# Supplementary material for: A network medicine approach to investigation and population-based validation of disease manifestations and drug repurposing for COVID-19
Source: PLoS Biol. 2020 Nov 6;18(11):e3000970. doi: 10.1371/journal.pbio.3000970 (PMC7728249; doi:10.1371/journal.pbio.3000970)
Supplement: S11 Fig — (A) UMAP visualization of non-epithelial cells from the ileal tissues of patients with Crohn disease. (B and D) The expression of ACE2 in the non-epithelial cells in (A). (C and E) The expression of TMPRSS2 in the non-epithelial cells in (A). (F) UMAP visualization of epithelial cells from the ileal tissues of patients with Crohn disease. (G and I) The expression of ACE2 in the epithelial cells in (F). (H and J) The expression of TMPRSS2 in the epithelial cells in (F). (K and L) The expression levels of ACE2 and TMPRSS2 in inflamed versus uninflamed ileal absorptive enterocytes in Crohn disease patients. The single-cell data were retrieved from Martin et al. [72], which contains 67,050 inflamed and uninflamed cells from the ileal samples of 8 patients with Crohn disease. (PDF) [file pbio.3000970.s022.pdf]

S11 Fig

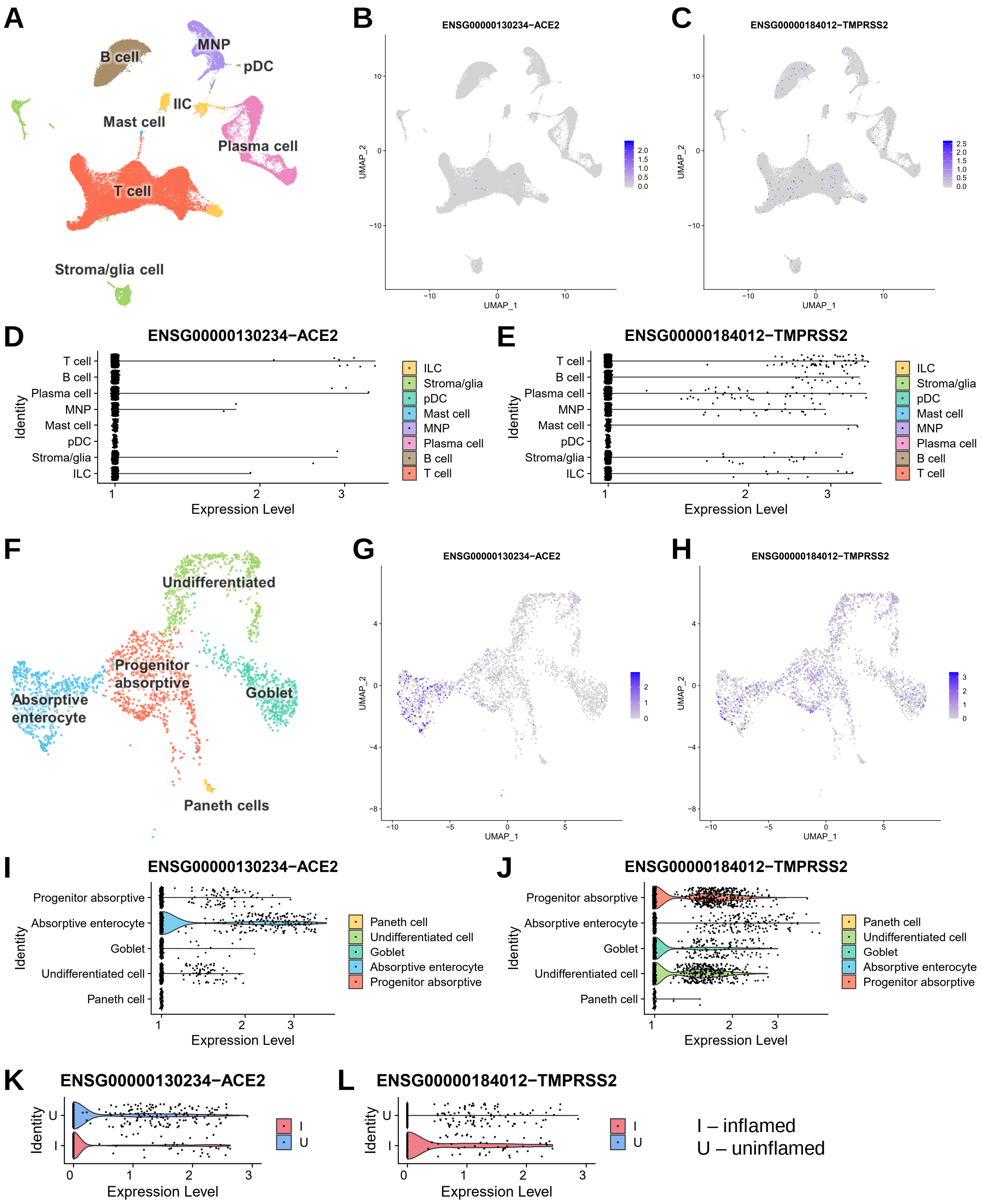

S11 Fig. Inflammatory bowel disease and COVID-19. (**A**) UMAP visualization of non-epithelial cells from the ileal tissues of patients with Crohn disease. (**B, D**) The expression of *ACE2* in the non-epithelial cells in **A**. (**C, E**) The expression of *TMPRSS2* in the non-epithelial cells in **A**. (**F**) UMAP visualization of epithelial cells from the ileal tissues of patients with Crohn disease. (**G, I**) The expression of *ACE2* in the epithelial cells in **F**. (**H, J**) The expression of *TMPRSS2* in the epithelial cells in **F**. (**K, L**) The expression levels of *ACE2* and *TMPRSS2* in inflamed versus uninflamed ileal absorptive enterocytes in Crohn disease patients. The single-cell data were retrieved from Martin, J. C. *et al*. [72], which contains 67,050 inflamed and uninflamed cells from the ileal samples of 8 patients with Crohn disease.
