# Supplementary material for: A network medicine approach to investigation and population-based validation of disease manifestations and drug repurposing for COVID-19
Source: PLoS Biol. 2020 Nov 6;18(11):e3000970. doi: 10.1371/journal.pbio.3000970 (PMC7728249; doi:10.1371/journal.pbio.3000970)
Supplement: S12 Fig — The disease subgroups are (A) asthma, (B) diabetes, and (C) hypertension. Four models were evaluated. These models were matched and adjusted using different variables, as shown in the table. The variable that was used to extract each patient subgroup was not used for propensity score matching or odds ratio adjustment. ACEI, angiotensin-converting enzyme inhibitor; ARB, angiotensin II receptor blocker. (PDF) [file pbio.3000970.s023.pdf]

S12 Fig

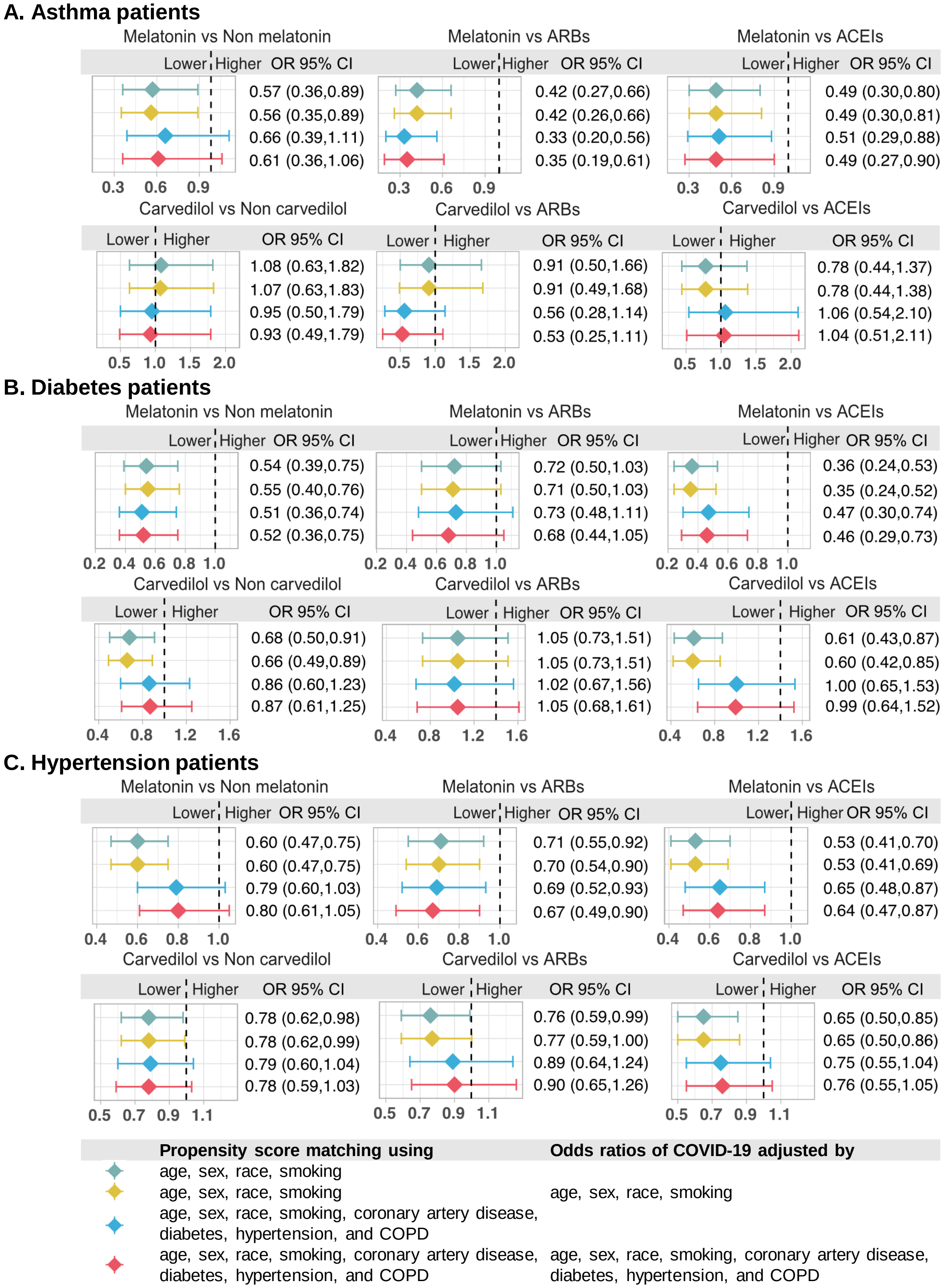

S12 Fig. Patient-based validation of drug repurposing for COVID-19 using three different disease subgroups. The disease subgroups are (**A**) asthma, (**B**) diabetes, and (**C**) hypertension. Four models were evaluated. These models were matched and adjusted using different variables as shown in the table. The variable that was used to extract each patient subgroup was not used for propensity score matching or odds ratio adjustment. ACEI, angiotensin-converting enzyme inhibitor. ARB, angiotensin II receptor blocker.
