# Supplementary material for: A network medicine approach to investigation and population-based validation of disease manifestations and drug repurposing for COVID-19
Source: PLoS Biol. 2020 Nov 6;18(11):e3000970. doi: 10.1371/journal.pbio.3000970 (PMC7728249; doi:10.1371/journal.pbio.3000970)
Supplement: S13 Fig — In black Americans, carvedilol use showed a lowered risk of a positive SARS-CoV-2 test when propensity score was matched with basic variables (age, sex, and smoking). (PDF) [file pbio.3000970.s024.pdf]

S13 Fig

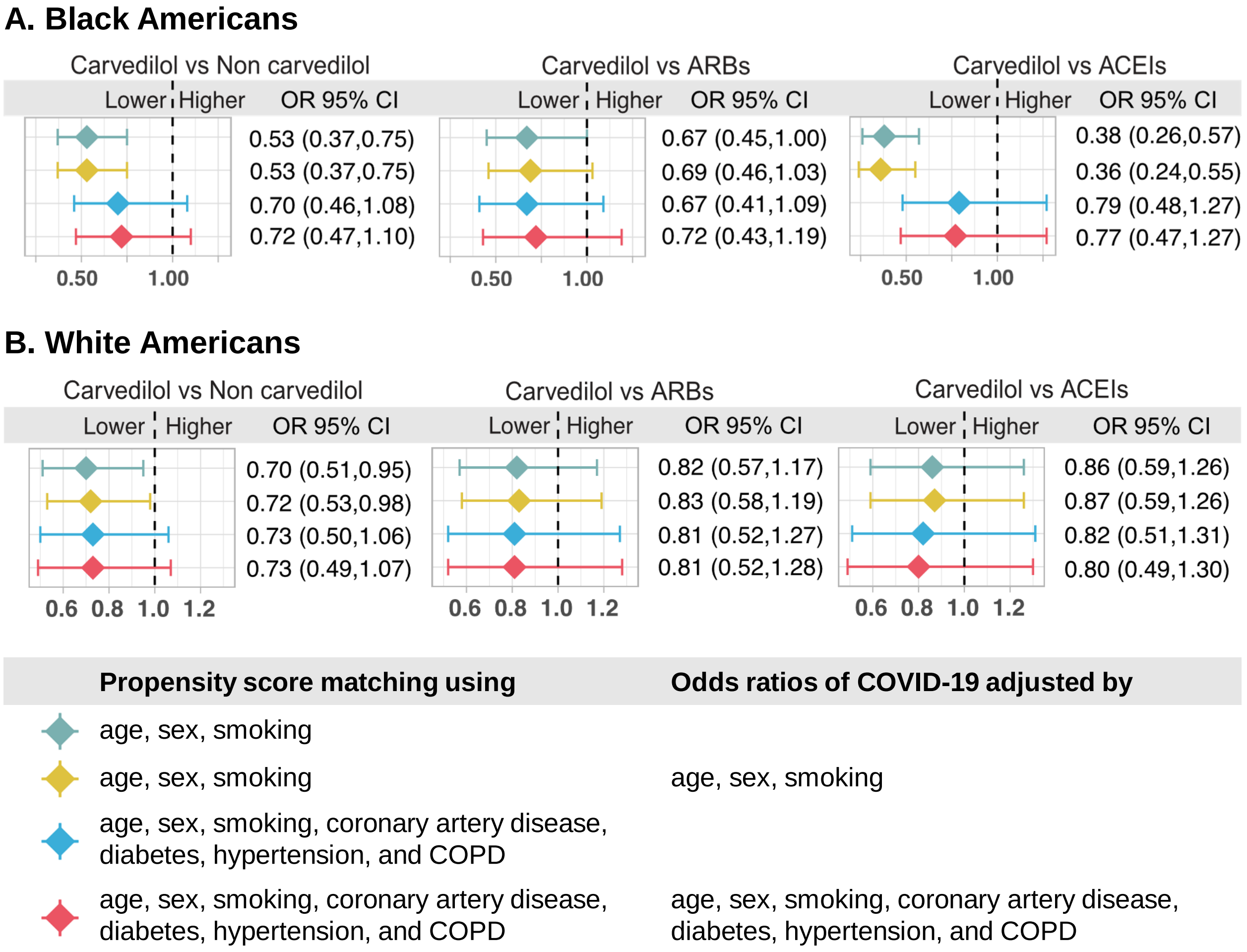

S13 Fig. Comparison of the patient validation results of carvedilol use in black Americans and white Americans. In black Americans, carvedilol use showed a lowered risk of a positive SARS-CoV-2 test when propensity score was matched with basic variables (age, sex, and smoking).
