# Supplementary material for: A network medicine approach to investigation and population-based validation of disease manifestations and drug repurposing for COVID-19
Source: PLoS Biol. 2020 Nov 6;18(11):e3000970. doi: 10.1371/journal.pbio.3000970 (PMC7728249; doi:10.1371/journal.pbio.3000970)
Supplement: S14 Fig — Each dot represents a disease (Z score versus number of genes). No significant bias was observed for the number of genes. The maximum R2 is 0.1468 from the SARS2-PPI dataset. The data underlying this figure can be found in S10 Data. (PDF) [file pbio.3000970.s025.pdf]

S14 Fig

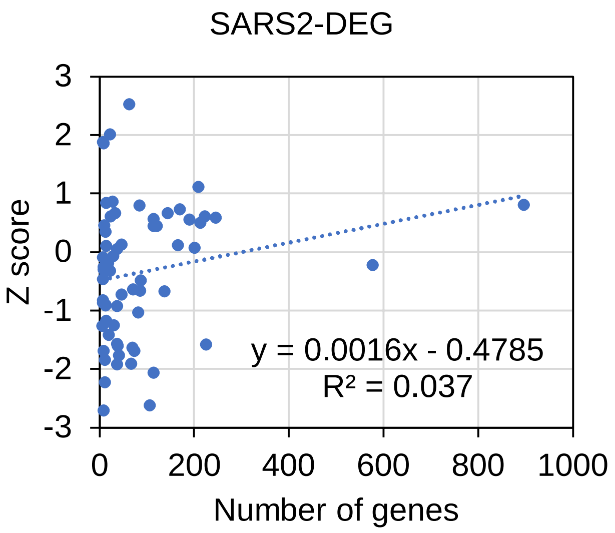

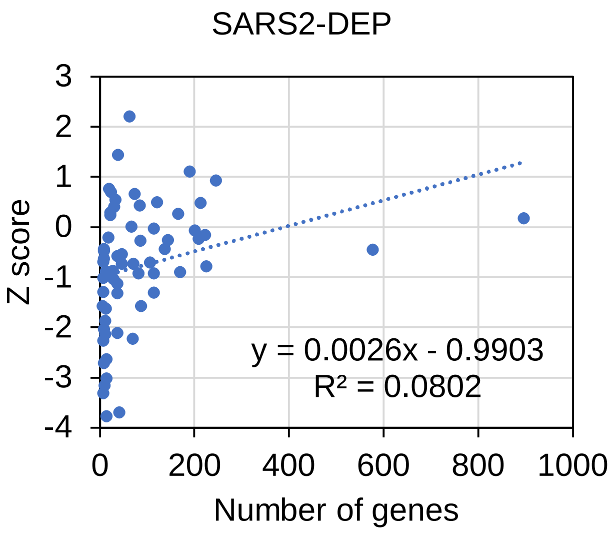

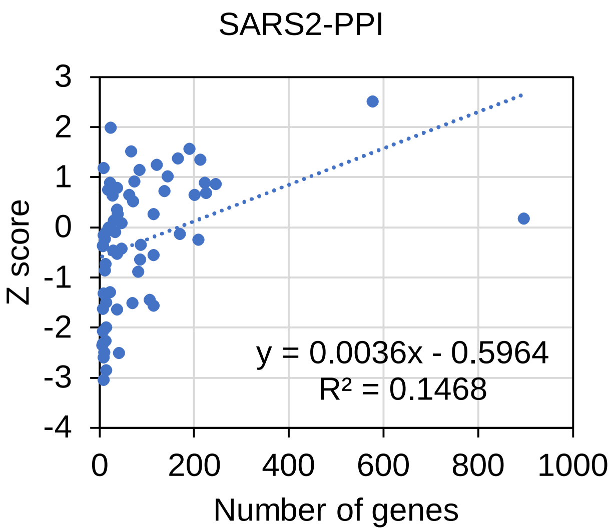

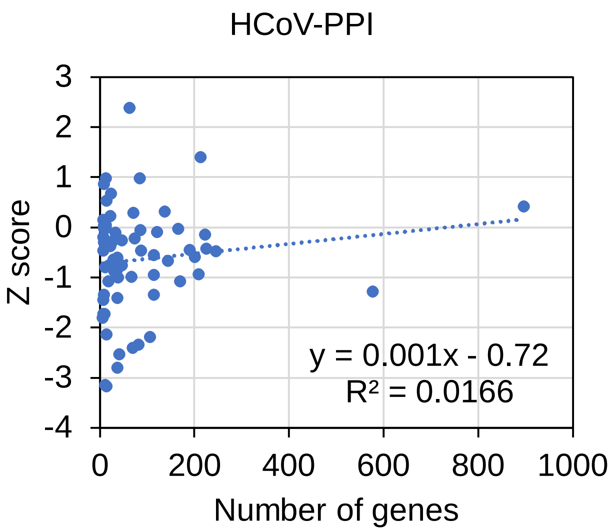

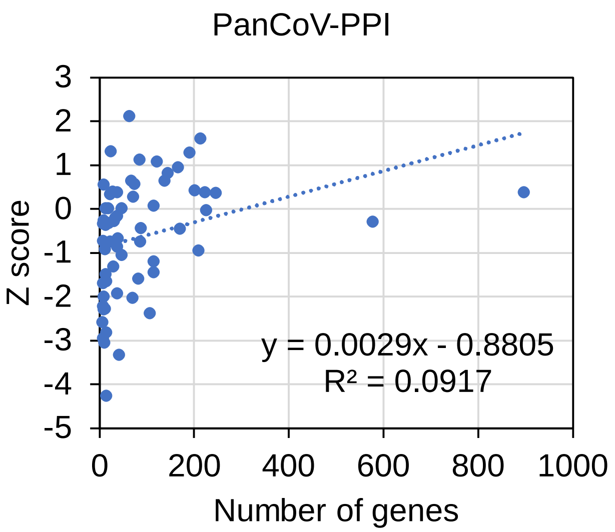

S14 Fig. Analysis of the effect of the number of genes associated with diseases on the network proximity z scores. Each dot represents a disease (z score versus number of genes). No significant bias was observed for the number of genes. The maximum R^2 is 0.1468 from the SARS2-PPI dataset. The data underlying this figure can be found in S10 Data.
