# Supplementary material for: A network medicine approach to investigation and population-based validation of disease manifestations and drug repurposing for COVID-19
Source: PLoS Biol. 2020 Nov 6;18(11):e3000970. doi: 10.1371/journal.pbio.3000970 (PMC7728249; doi:10.1371/journal.pbio.3000970)
Supplement: S15 Fig — The disease-associated genes were filtered by their tissue specificity. Tissues considered are shown after the disease names. Only genes with positive specificity were retained for the network analysis. After filtering, diseases with fewer than 5 genes were removed from the evaluation. The data underlying this figure can be found in S11 Data. (PDF) [file pbio.3000970.s026.pdf]

S15 Fig

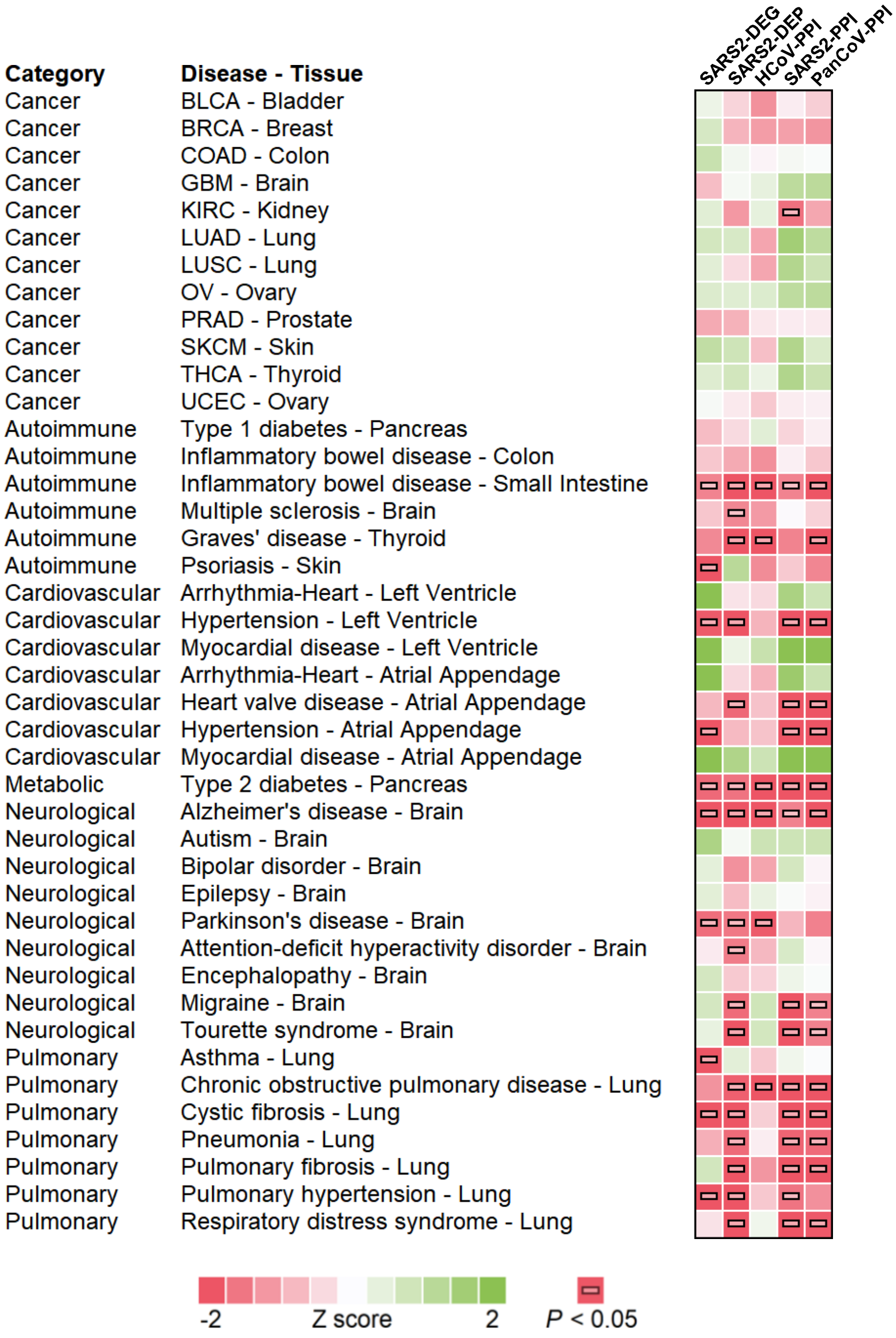

S15 Fig. Disease manifestations associated with COVID-19 quantified by network proximity measurement using tissue-specific genes for each disease. The disease-associated genes were filtered by their tissue specificity. Tissues considered are shown after the disease names. Only genes with positive specificity were retained for the network analysis. After filtering, diseases with fewer than 5 genes were removed from the evaluation. The data underlying this figure can be found in S11 Data.
