# Supplementary material for: A network medicine approach to investigation and population-based validation of disease manifestations and drug repurposing for COVID-19
Source: PLoS Biol. 2020 Nov 6;18(11):e3000970. doi: 10.1371/journal.pbio.3000970 (PMC7728249; doi:10.1371/journal.pbio.3000970)
Supplement: S16 Fig — Node sizes show their tissue specificity in colon. (PDF) [file pbio.3000970.s027.pdf]

S16 Fig

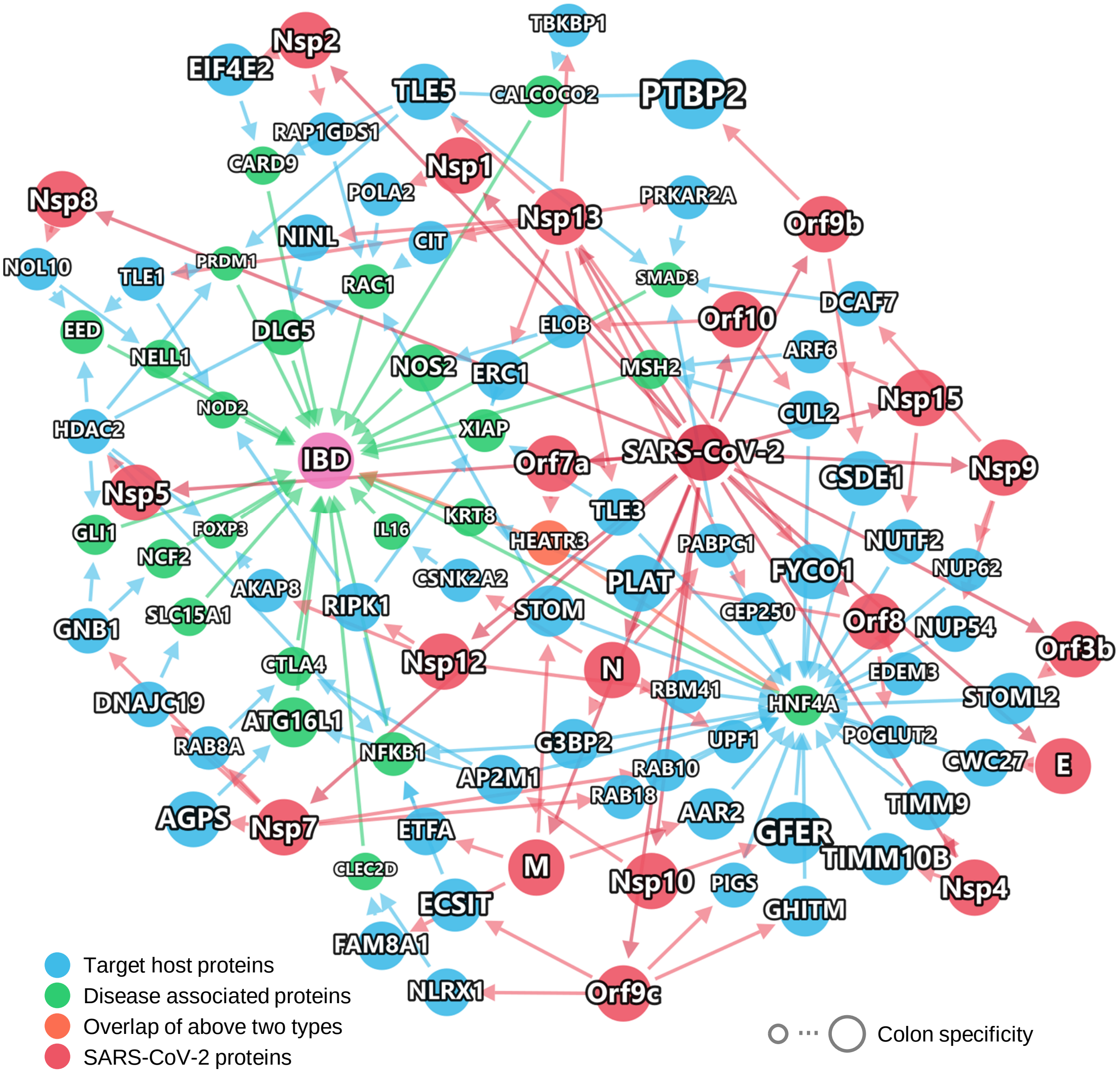

S16 Fig. The subnetwork between the IBD-associated genes, the SARS-CoV-2 virus proteins, and virus target proteins. Node sizes show their tissue specificity in colon.
