# Supplementary material for: A network medicine approach to investigation and population-based validation of disease manifestations and drug repurposing for COVID-19
Source: PLoS Biol. 2020 Nov 6;18(11):e3000970. doi: 10.1371/journal.pbio.3000970 (PMC7728249; doi:10.1371/journal.pbio.3000970)
Supplement: S17 Fig — The up- and down-expressed genes in the 2 asthma datasets (GSE63142 and GSE130499, severe versus control) were computed against the up- and down-expressed genes from the SARS2-DEG dataset. Overall, the results show more significant network proximities and smaller Z scores than when the direction is not considered, as in Fig 4. (PDF) [file pbio.3000970.s028.pdf]

S17 Fig

Z score		SARS2-DEG Up DEG	SARS2-DEG Down DEG
GSE63142	Up DEG	-1.3	-0.2
	Down DEG	-1.5	-1.7
GSE130499	Up DEG	-1.2	0.2
	Down DEG	-2.8	-1.7

P value		SARS2-DEG Up DEG	SARS2-DEG Down DEG
GSE63142	Up DEG	0.08	0.38
	Down DEG	0.05	* 0.04
GSE130499	Up DEG	0.07	0.52
	Down DEG	* 0.01	* 0.04

-2 Z score 2

* P < 0.05

S17 Fig. Network proximity analysis of asthma and COVID-19 taking into consideration the directionalities of the differential gene expression. The up- and down- expressed genes in the two asthma datasets (GSE63142 and GSE130499, severe vs. control) were computed against the up- and down- expressed genes from the SARS2-DEG dataset. Overall, the results show more significant network proximities and smaller z scores than when the direction is not considered, as in Fig 4.
