# Supplementary material for: A network medicine approach to investigation and population-based validation of disease manifestations and drug repurposing for COVID-19
Source: PLoS Biol. 2020 Nov 6;18(11):e3000970. doi: 10.1371/journal.pbio.3000970 (PMC7728249; doi:10.1371/journal.pbio.3000970)
Supplement: S18 Fig — We searched PubMed, Embase, and medRxiv databases for publications as of April 25, 2020, using the search term (“SARS-COV-2” OR “COVID-19” OR “nCoV 19” OR “2019 novel coronavirus” OR “coronavirus disease 2019”) AND (“clinical characteristics” OR “clinical outcome” OR “comorbidities”). Only research articles were included. Several criteria were used to filter the initial 1,054 articles to a final sample of 34 studies for meta-analyses. (PDF) [file pbio.3000970.s029.pdf]

S18 Fig

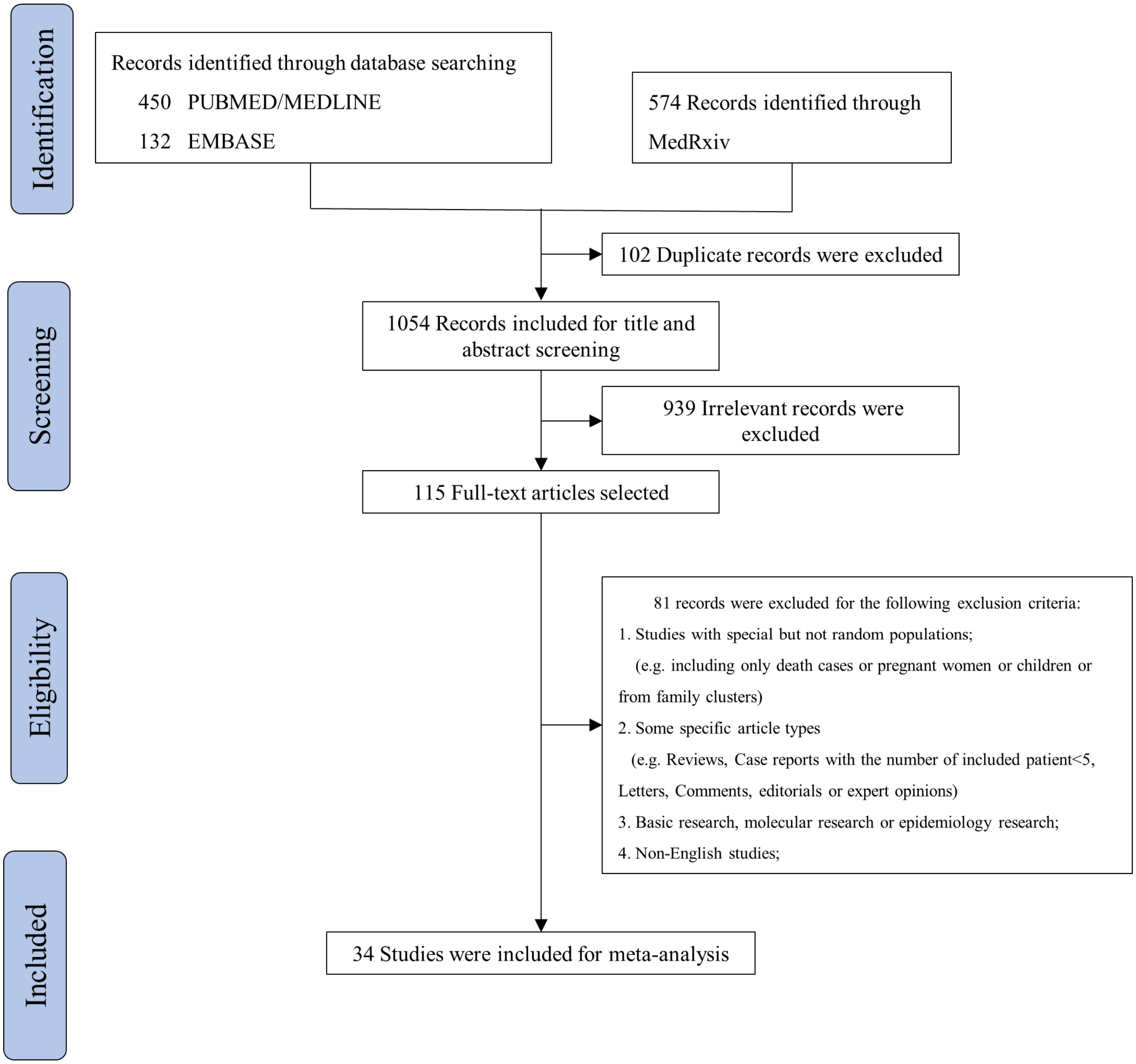

S18 Fig. Workflow of clinical study search. We searched PubMed, Embase, and medRxiv databases for publications as of April 25th, 2020 using the search term (“SARS-COV-2” OR “COVID-19” OR “nCoV 19” OR “2019 novel coronavirus” OR “coronavirus disease 2019”) AND (“clinical characteristics” OR “clinical outcome” OR “comorbidities”). Only research articles were included. Several criteria were used to filter the initial 1,054 articles to a final sample of 34 studies for meta-analyses.
