# Supplementary material for: A network medicine approach to investigation and population-based validation of disease manifestations and drug repurposing for COVID-19
Source: PLoS Biol. 2020 Nov 6;18(11):e3000970. doi: 10.1371/journal.pbio.3000970 (PMC7728249; doi:10.1371/journal.pbio.3000970)
Supplement: S19 Fig — Cytoscape 3.7.1 was used for the visualization and for generating the statistics. Clustering coefficient (ranges from 0 to 1) measures the extent to which the nodes in the network tend to cluster together. Network centralization (ranges from 0 to 1) measures the extent to which the topology resembles a star. Network density (ranges from 0 to 1) shows how densely the nodes are connected in the network. Network heterogeneity shows the tendency of the network to contain hub nodes. (PDF) [file pbio.3000970.s030.pdf]

S19 Fig

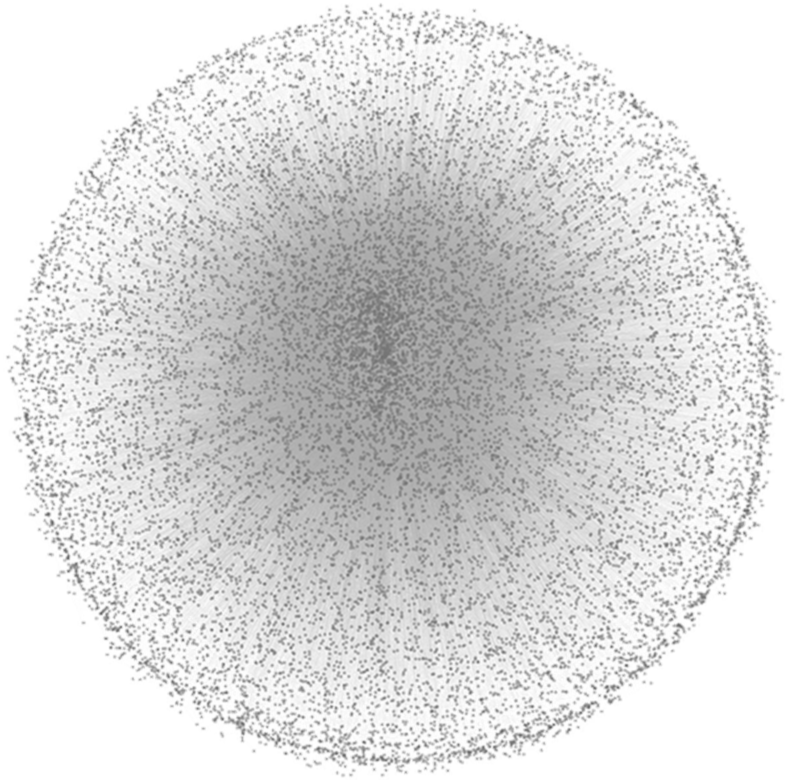

Number of nodes	17706
Number of edges	351444
Network density	0.002
Degree (min-mean-max)	1 - 39.7 - 9777
Distance (min-mean-max)	1 - 2.70 - 8
Clustering coefficient	0.158
Network centralization	0.550
Network density	0.002
Network heterogeneity	2.775

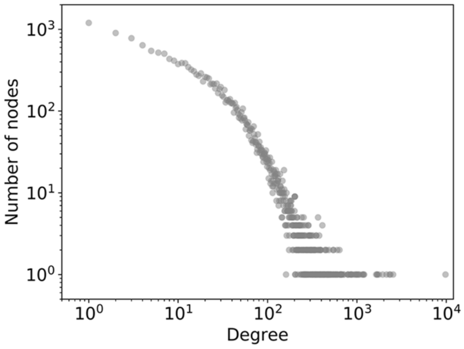

S19 Fig. Overview of the human protein interactome. Cytoscape 3.7.1 was used for the visualization and for generating the statistics. Clustering coefficient (ranges from 0 to 1) measures the extent to which the nodes in the network tend to cluster together. Network centralization (ranges from 0 to 1) measures the extent to which the topology resembles a star. Network density (ranges from 0 to 1) shows how densely the nodes are connected in the network. Network heterogeneity shows the tendency of the network to contain hub nodes.
