# Supplementary material for: A network medicine approach to investigation and population-based validation of disease manifestations and drug repurposing for COVID-19
Source: PLoS Biol. 2020 Nov 6;18(11):e3000970. doi: 10.1371/journal.pbio.3000970 (PMC7728249; doi:10.1371/journal.pbio.3000970)
Supplement: S20 Fig — (PDF) [file pbio.3000970.s031.pdf]

S20 Fig

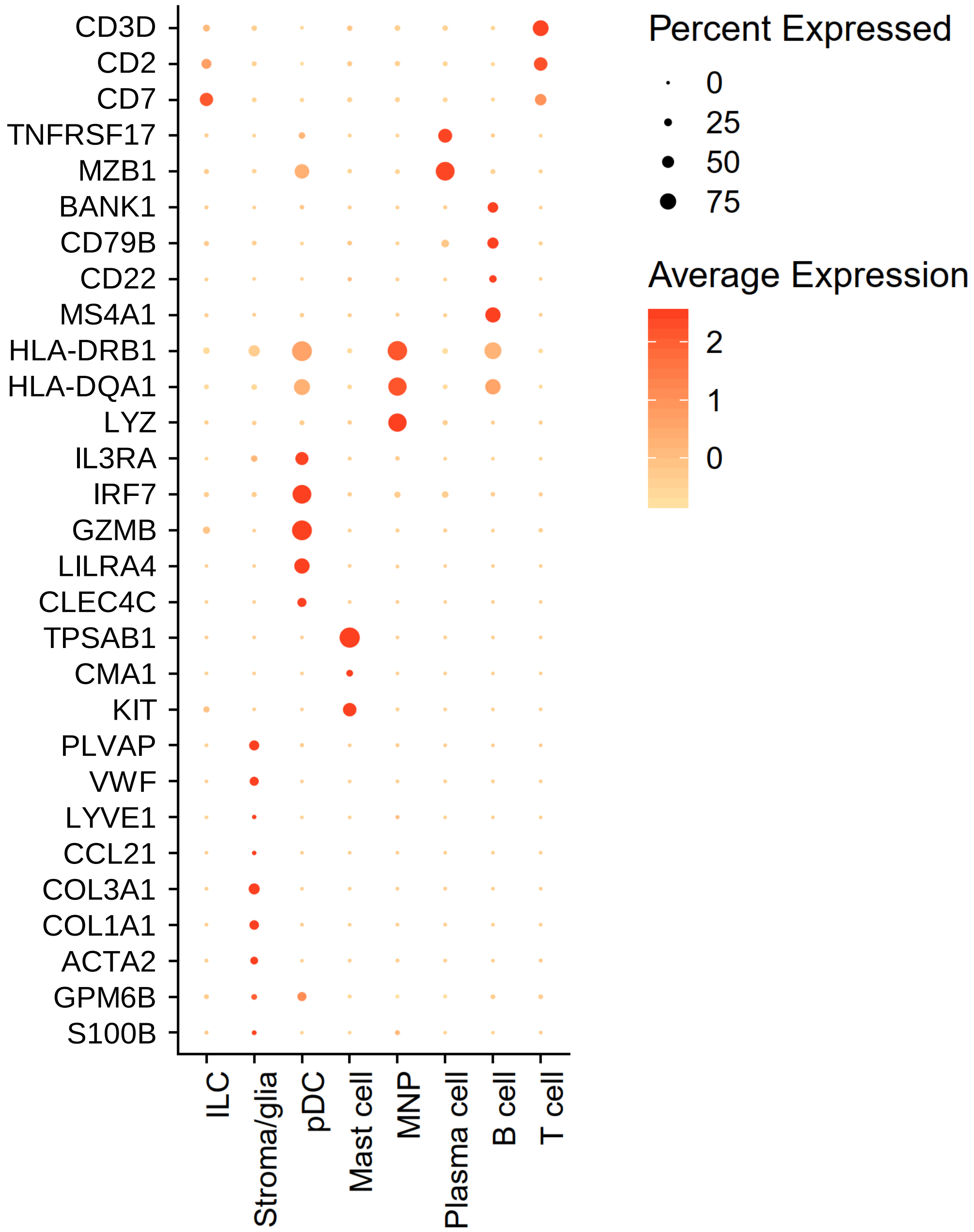

S20 Fig. Cell type markers and their expressions in dot plot used to identify the ileal non-epithelial cells.
