# Supplementary material for: A network medicine approach to investigation and population-based validation of disease manifestations and drug repurposing for COVID-19
Source: PLoS Biol. 2020 Nov 6;18(11):e3000970. doi: 10.1371/journal.pbio.3000970 (PMC7728249; doi:10.1371/journal.pbio.3000970)
Supplement: S1 Table — (PDF) [file pbio.3000970.s034.pdf]

S1 Table. Summary of the data sets used in this study.

Data set	Description	PubMed ID and/or GEO
Blanco-Melo D *et al.*,<br>*Cell.* 2020	Alias: SARS2-DEG<br>Purpose: Drug screen and comorbidity identification, GSEA<br>Groups: Human bronchial epithelial cells infected with SARS-CoV-2 (3 replicates) vs. uninfected (3 replicates)	32416070<br>GSE147507
Bojkova D *et al.*,<br>*Nature.* 2020	Alias: SARS2-DEP<br>Purpose: Drug screen and comorbidity identification, GSEA<br>Groups: Human Caco-2 cells infected with SARS-CoV-2 vs. uninfected	32408336
Zhou Y *et al.*,<br>*Cell Discov.* 2020	Alias: HCoV-PPI<br>Purpose: Drug screen and comorbidity identification<br>134 strong literature evidence-based pan-human coronavirus target host proteins including 15 newly added proteins	32194980
Gordon DE *et al.*,<br>*Nature.* 2020.	Alias: SARS2-PPI<br>Purpose: Drug screen and comorbidity identification<br>332 proteins involved in the protein-protein interactions with 26 SARS-CoV-2 viral proteins identified by affinity purification-mass spectrometry	32353859
Modena BD *et al.*,<br>*Am J Respir Crit Care Med.* 2014	Purpose: To provide DEGs in asthma patients<br>bronchial epithelial cells of 27 control samples, 72 mild asthma samples, and 56 severe asthma samples were obtained by bronchoscopy with endobronchial epithelial brushing	25338189<br>GSE63142
Weathington N *et al.*,<br>*Am J Respir Crit Care Med.* 2019	Purpose: To provide DEGs in asthma patients<br>bronchial epithelial cells of 38 control samples, 72 mild asthma samples, and 44 severe asthma samples were available by bronchoscopy with endobronchial epithelial brushing	31161938<br>GSE130499
Lukassen S *et al.*,<br>*EMBO J.* 2020	Purpose: Single-cell study for understanding the association between COVID-19 and asthma<br>This data set contains single-cell data of normal lung and primary human bronchial epithelial cells	32246845
Martin JC *et al.*,<br>*Cell.* 2019	Purpose: Single-cell study for understanding the association between COVID-19 and IBD<br>This data set contains both inflamed and uninflamed cells from the ileal samples of 8 patients with Crohn’s disease	31474370<br>GSE134809
Yuan S *et al.*,<br>*Nat Commun.* 2019	Alias: MERS<br>Purpose: To provide DEGs for GSEA<br>Groups: MERS-CoV infected Calu-3 cells (3 replicates) vs. uninfected (3 replicates)	30631056<br>GSE122876
Sims AC *et al.*,<br>*J Virol.* 2013	Alias: SARSa<br>Purpose: To provide DEGs for GSEA<br>Groups: SARS-CoV-1 infected Calu-3 cells (3 replicates) vs. uninfected (3 replicates)	23365422<br>GSE33267
Reghunathan R *et al.*,<br>*BMC Immunol.* 2005	Alias: SARSa<br>Purpose: To provide DEGs for GSEA<br>Groups: SARS-CoV-1 infected patients’ peripheral blood (10 samples) vs. normal blood samples (4 samples)	15655079<br>GSE1739

GSEA, Gene set enrichment analysis. DEG, differentially expressed gene. DEP, differentially expressed protein. PPI, protein-protein interaction. IBD, inflammatory bowel disease.
