# Supplementary material for: A network medicine approach to investigation and population-based validation of disease manifestations and drug repurposing for COVID-19
Source: PLoS Biol. 2020 Nov 6;18(11):e3000970. doi: 10.1371/journal.pbio.3000970 (PMC7728249; doi:10.1371/journal.pbio.3000970)
Supplement: Attachment — Submitted filename: renamed_9304e.pdf [file pbio.3000970.s040.pdf]

Response to Editor's comments.

Your Manuscript file is over our file size limit of 10MB. This limit is in place for the convenience of reviewers, editors and readers. Please adapt this file so that the file size is below 10MB.

Response: We have reduced the total size of Manuscript files (main text and Figures 1-7) less than 10 MB.
