# Supplementary material for: A network medicine approach to investigation and population-based validation of disease manifestations and drug repurposing for COVID-19
Source: PLoS Biol. 2020 Nov 6;18(11):e3000970. doi: 10.1371/journal.pbio.3000970 (PMC7728249; doi:10.1371/journal.pbio.3000970)
Supplement: Attachment — Submitted filename: Response_to_Reviewers_Sep2.pdf [file pbio.3000970.s041.pdf]

Manuscript #: PBIOLOGY-D-20-01653R1

Dear Dr. Roberts and Reviewers,

Thank you for your time and thoughtful feedback. We have crafted the itemized responses to your comments as below.

Responses to Reviewer #1

Zhou et al developed network-based methodologies to identify SARS-CoV-2 pathogenesis, disease manifestations, and COVID-19 therapies. They incorporated SARSCoV-2 virus-host protein-protein interactions, transcriptomics, and proteomics into the human interactome. Network proximity measure was used to identify the underlying pathogenesis for broad COVID-19-associated manifestations. Multi-modal analyses of single-cell RNA-seq data identified the co-expression pattern of ACE2 and TMPRSS2 in absorptive enterocytes from the inflamed ileal tissues of Crohn's disease patients compared to uninflamed tissues, revealing shared pathobiology by COVID-19 and inflammatory bowel disease. Integrative analyses of metabolomics and transcriptomics (bulk and single-cell) data from asthma patients indicated that COVID-19 shared intermediate inflammatory endophenotypes with asthma. By combing network-based prediction and propensity score matching observation study of 18118 patients from a COVID-19 registry, the authors identified that melatonin was associated with 64% reduced likelihood of a positive lab test for SARS-CoV-2 and can have better efficacy than angiotensin II receptor blockers or angiotensin converting enzyme inhibitors for treating SARS-CoV-2. However, details about how to identify the differential expressed genes, PPI-networks and drug-target network construction, and single-cell RNA-seq analysis are missing; the rationale about the construction of PanCoV-PPI and the network proximity measure need to be clarified. Most of the results are computational discoveries. It may provide valuable insight if the following concerns are addressed.

Response: We thank the Reviewer for the great summary and constructive comments. We have intensified the rigor of our computational analysis and performed new experiments to meet the criticisms raised.

1. Details about the RNA-seq data analysis are missing. I am not sure how the authors generated SARS2-DEP. For example, which samples in GSE147507 are used to identify differentially expressed genes? Which samples are used as controls? What kind of software is used to analyze those samples? How to identify differentially expressed genes? The same problem arises elsewhere in the manuscript.

Response: We added **S1 Table** for a list of all the data sets used in this study. We have also added more details for the bioinformatics processing of each data set:

(page 35) "***SARS2-DEG.*** *In the original study, the primary human bronchial epithelial cells were infected with SARS-CoV-2 for 24 hours. The transcriptome profiles of infected (3 replicates) and uninfected cells (3 replicates) were characterized, and the fold change (FC) and false discovery rate (FDR) for each gene were calculated by DESeq2 and provided in the original study. We applied a cutoff of |logFC| > 0.5 and FDR < 0.05 to identify the differentially expressed genes.*

SARS2-DEP. *As described in the previous study, human Caco-2 cells were infected with SARS-CoV-2 for up to 24 hours. Proteomics assays of the infected and uninfected cells were measured at 24 hours in triplicates. We used the results at 24 hours, as the original study showed most differentially expressed proteins at 24 hours. The P values were computed using two-sided unpaired Student's t-test with equal variance assumed in this study. We converted the P value to FDR using the "fdrcorrection" function from the Python package statsmodels v0.11.1 and used a cutoff of FDR < 0.05 to identify the differentially expressed proteins.*"

(page 42) "*Differential expression of three comparisons, severe vs. control, mild vs. control, and severe vs. mild were performed using the GEO2R function (https://www.ncbi.nlm.nih.gov/geo/geo2r/) [135]. In GSE63142 [64], bronchial epithelial cells of 27 control samples, 72 mild asthma samples, and 56 severe asthma samples were obtained by bronchoscopy with endobronchial epithelial brushing. In GSE130499 [65], bronchial epithelial cells of 38 control samples, 72 mild asthma samples, and 44 severe asthma samples were available by bronchoscopy with endobronchial epithelial brushing as well. The differential expression analysis was performed by defining the groups in GEO2R first, then by selecting the two groups to compare. Genes with |logFC| > 0.5 and FDR < 0.05 were considered significantly differentially expressed.*"

Reference for Comment #1

[135] Barrett T, Wilhite SE, Ledoux P, Evangelista C, Kim IF, Tomashevsky M, et al. NCBI GEO: archive for functional genomics data sets--update. *Nucleic Acids Res.* 2013;41(Database issue):D991-5. Epub 2012/11/30. doi: 10.1093/nar/gks1193.

2. In line 194 of page 10, the authors mentioned that "For cancer, the driver genes for pan-cancer and individual cancer types were retrieved from the Cancer Gene Census [34] and a previous study [35]. For autoimmune, pulmonary, neurological, cardiovascular, and metabolic categories, we extracted their associated genes/proteins from the Human Gene Mutation Database" I am not sure how those genes were retrieved from corresponding databases? Did the authors use any keywords for searching?

Response: For somatic driver genes in cancer, we defined a driver gene if a gene had significantly enriched driver mutations based on whole-genome or whole-exome sequencing data or reported experimental data from the Cancer Gene Census (Sondka et al., *Nature Review Cancer* 2018) or the original publications from The Cancer Genome Atlas (TCGA, https://portal.gdc.cancer.gov).

For autoimmune, pulmonary, neurological, cardiovascular, and metabolic categories from the Human Gene Mutation Database (HGMD) (Stenson et al., *Human Mutation* 2003), we defined a disease-associated gene if a gene has at least one disease-associated mutation in original publications provided in HGMD. HGMD is a well-documented disease gene database and we downloaded the whole database for data analysis and extraction by using the well-documented disease ontology terms (Bello et al., *Dis Model Mech* 2018). Thus, we don't need to use keywords from HGMD database interface search as it may generate data incompleteness as a disease has multiple Ontology terms.

We have added more details in **S4 Table**, including the data sources, number of genes, mutations associated with the disease, and terms for identifying diseases in the HGMD. We also added these details in page 36 of the revised manuscript.

References for Comment #2

1. Sondka Z, Bamford S, Cole CG, Ward SA, Dunham I, Forbes SA. The COSMIC Cancer Gene Census: describing genetic dysfunction across all human cancers. *Nat Rev Cancer.* 2018;18(11):696-705. Epub 2018/10/08. doi: 10.1038/s41568-018-0060-1.

2. Stenson PD, Ball EV, Mort M, Phillips AD, Shiel JA, Thomas NS, et al. Human Gene Mutation Database (HGMD): 2003 update. *Hum Mutat.* 2003;21(6):577-81. Epub 2003/05/20. doi: 10.1002/humu.10212.

3. Bello SM, Shimoyama M, Mitraka E, Laulederkind SJF, Smith CL, Eppig JT, et al. Disease Ontology: improving and unifying disease annotations across species. *Dis Model Mech.* 2018;11(3). Epub 2018/03/29. doi: 10.1242/dmm.032839.

3. In line 675 of page 31, the authors mentioned that "The final human protein-protein interactome used in this study included 351,444 unique PPIs connecting 17,706 proteins". However, would the authors put those results in their GitHub page for the reader's reference? Several methods adopted are described as "as described in our previous study". Some statistics/overview of the network features/methods, although published somewhere else, should be provided for a better understanding of the proposed methods. For example, how did the authors build human protein-protein interactome?

Response: We have uploaded the human protein-protein interactome to the GitHub page. The interactome was built by combining PPIs with one of the five types of evidences from 18 bioinformatics databases or published resources as mentioned in the Method section: **Building the human protein-protein interactome** (page 40). The source and experimental evidence type of each PPI can be found in the revised Methods section and the uploaded file in the GitHub page (https://github.com/ChengF-Lab/COVID-19_Map). We have also added basic statistics and a visualization of the human protein interactome in **S19 Fig** and **Extended Figure 1**.

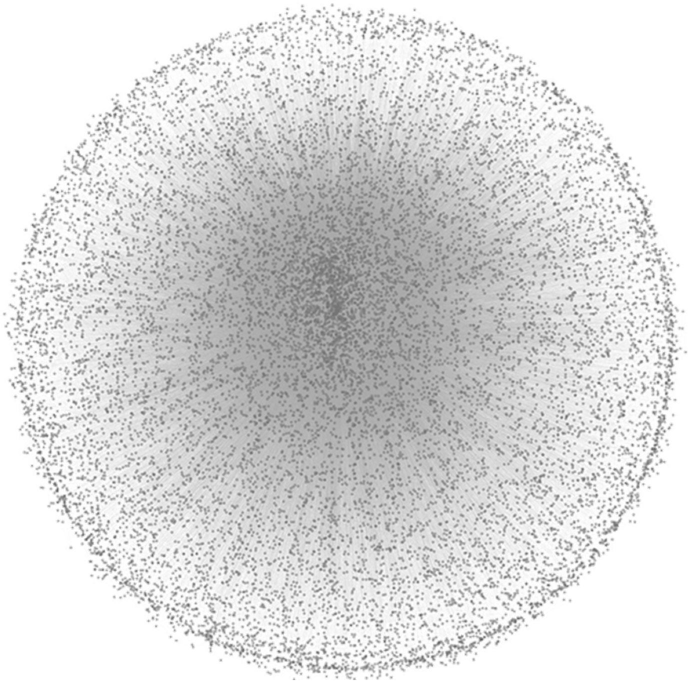

Number of nodes	17706
Number of edges	351444
Network density	0.002
Degree (min-mean-max)	1 - 39.7 - 9777
Distance (min-mean-max)	1 - 2.70 - 8
Clustering coefficient	0.158
Network centralization	0.550
Network density	0.002
Network heterogeneity	2.775

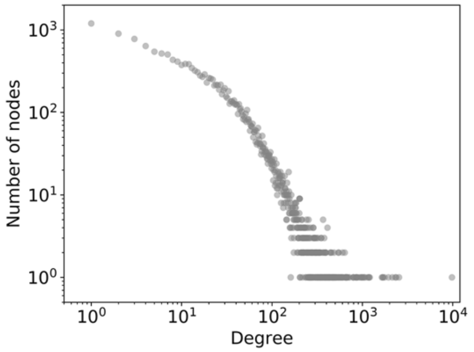

Extended Figure 1. Overview of the human protein interactome. Cytoscape 3.7.1 was used for the visualization and for generating the statistics. Clustering coefficient (ranges from 0 to 1) measures the extent to which the nodes in the network tend to cluster together. Network centralization (ranges from 0 to 1) measures the extent to which the topology resembles a star. Network density (ranges from 0 to 1) shows how densely the nodes are connected in the network. Network heterogeneity shows the tendency of the network to contain hub nodes.

4. What are active comparator design and PS adjustment?

Response: Active comparator design is a state-of-the-art pharmacoepidemiologic analysis to validate the drug-disease outcome using electronic patient data, as described in our previous study (Cheng et al., *Nature Communications* 2018). For active comparator design, we used angiotensin II receptor blockers (ARBs) or angiotensin-converting enzyme inhibitors (ACEIs) as comparators, as both ARBs and ACEIs were not associated with risk of SARS-CoV-2 infection in several recent studies (Vaduganathan et al., *N Engl J Med* 2020; Jarcho et al., *N Engl J Med* 2020; Mehta et al., *JAMA Cardiology* 2020). In addition, a recent study showed that inpatient use of ACEI/ARB was associated with lower risk of all-cause mortality compared with ACEI/ARB non-users hospitalized COVID-19 patients with hypertension (Zhang et al., *Circ Res.* 2020). Altogether, these reports provide evidence that ARBs and ACEIs are

gold-standard comparators for propensity score (PS)-matched active comparator design studies as described (Cheng et al., *Nature Commun* 2018). We used propensity score (PS) to adjust for age, sex, race, smoking history, and various disease comorbidities (coronary artery disease, diabetes, hypertension, and COPD) during all drug-outcome analysis, including active comparator design observations.

References for Comment #4

1. Vaduganathan M, Vardeny O, Michel T, McMurray JJV, Pfeffer MA, Solomon SD. Renin-Angiotensin-Aldosterone System Inhibitors in Patients with Covid-19. *N Engl J Med.* 2020;382(17):1653-9. Epub 2020/04/01. doi: 10.1056/NEJMsr2005760.

2. Jarcho JA, Ingelfinger JR, Hamel MB, D'Agostino RB, Sr., Harrington DP. Inhibitors of the Renin-Angiotensin-Aldosterone System and Covid-19. *N Engl J Med.* 2020;382(25):2462-4. Epub 2020/05/02. doi: 10.1056/NEJMe2012924.

3. Mehta N, Kalra A, Nowacki AS, Anjewierden S, Han Z, Bhat P, et al. Association of Use of Angiotensin-Converting Enzyme Inhibitors and Angiotensin II Receptor Blockers With Testing Positive for Coronavirus Disease 2019 (COVID-19). *JAMA Cardiol.* 2020. Epub 2020/05/06. doi: 10.1001/jamacardio.2020.1855.

4. Zhang P, Zhu L, Cai J, Lei F, Qin JJ, Xie J, et al. Association of Inpatient Use of Angiotensin-Converting Enzyme Inhibitors and Angiotensin II Receptor Blockers With Mortality Among Patients With Hypertension Hospitalized With COVID-19. *Circ Res.* 2020;126(12):1671-81. Epub 2020/04/18. doi: 10.1161/CIRCRESAHA.120.317134.

5. Cheng F, Desai RJ, Handy DE, Wang R, Schneeweiss S, Barabasi AL, et al. Network-based approach to prediction and population-based validation of in silico drug repurposing. *Nat Commun.* 2018;9(1):2691. Epub 2018/07/14. doi: 10.1038/s41467-018-05116-5.

5. How did the authors obtain the experimentally validated drug-target network?

Response: We collected physical drug–target interactions from six sources, including DrugBank, Therapeutic Target Database, PharmGKB, ChEMBL, BindingDB, and IUPHAR/BPS Guide to PHARMACOLOGY. We only retained those drug-protein pairs where the binding affinities (including K_i, K_d, IC_{50}, or EC_{50}) were ≤ 10 μM and the human protein has unique "reviewed" accession number in the UniProt database. This strategy of building experimentally validated drug-target network has been widely used in our previous studies (Cheng et al., *Nature Communications* 2018, 2019a and 2019b).

References for Comment #5

Cheng F, Desai RJ, Handy DE, Wang R, Schneeweiss S, Barabasi AL, et al. Network-based approach to prediction and population-based validation of in silico drug repurposing. *Nat Commun.* 2018;9(1):2691. Epub 2018/07/14. doi: 10.1038/s41467-018-05116-5.

Cheng F, Kovacs IA, Barabasi AL. Network-based prediction of drug combinations. *Nat Commun.* 2019;10(1):1197. Epub 2019/03/15. doi: 10.1038/s41467-019-09186-x.

Cheng F, Lu W, Liu C, Fang J, Hou Y, Handy DE, et al. A genome-wide positioning systems network algorithm for in silico drug repurposing. *Nat Commun.* 2019;10(1):3476. Epub 2019/08/04. doi: 10.1038/s41467-019-10744-6.

6. Source code and supporting data cannot be found from the Github link provided by the authors.

Response: We apologize that the GitHub link was not shown correctly in the original manuscript. We have uploaded the code and supporting data to the GitHub page: https://github.com/ChengF-Lab/COVID-19_Map

7. In line 138-142, the authors mentioned that "PanCoV-PPI (Fig. 2B) and four other data sets (SARS2-139 DEG, SARS2-DEP, HCoV-PPI, and SARS2-PPI) (S6 Fig) were more likely to be highly connected (high degree or connectivity) in the human PPI network, including several hubs, such as JUN, XPO1, MOV10, NPM1, VCP, and HNRNPA1". According to Fig. 2B and S6, I cannot see anything supporting data to explain that JUN, XPO1, MOV10, NPM1, VCP, and HNRNPA1 are the hubs.

Response: We have revised the Supplemental tables (**S2 Table**) of the genes from the five SARS-CoV-2 gene/protein sets to include their Entrez ID, symbol, lung expression specificity, degree (connectivity) in the human interactome, degree in the subnetwork of this data set, *dN/dS* ratio, and evolutionary rate ratio. We changed the original text to "*Several hub genes, such as JUN, XPO1, MOV10, NPM1, VCP, and HNRNPA1, have the highest degree (connectivity) in the PanCoV-PPI network (S2 Table).*" in pages 8 and 9 of the revised manuscript.

8. The authors performed functional enrichment analyses for five different PPIs, i.e., SARS2-DEG, SARS2-DEP, HCoV-PPI, SARS2-PPI, and PanCoV-PPI, generated from transcriptomic and proteomic data of SARS-CoV-2 as well as literature-based virus-host protein-protein interactions. They found different PPIs differ considerably in terms of

enriched pathways, and then claimed "These observations suggest that these different SARS-CoV-2 data sets capture complementary aspects of the biological and cellular states of the viral life cycle and host immunity". However, many factors can cause the complementary effects. For example, 1. These data are derived from different cells or tissues, and not representative.

Response: We utilized SARS-CoV-2 virus-host PPIs and differentially expressed genes/proteins derived from different cells or tissues, as single cell lines have limitations for COVID-19 drug testing. For example, two recent studies suggested that chloroquine or hydroxychloroquine showed ideal antiviral activities in African green monkey kidney cells (VeroE6) but not in *a model of reconstituted human airway epithelium or TMPRSS2-positive lung cell line Calu-3* (Maisonnasse et al., *Nature* 2020; Hoffmann et al., *Nature* 2020). These studies showed that cell lines mimicking important aspects of respiratory epithelial cells should be used when analyzing the antiviral activity of drugs targeting host cell functions.

Although SARS2-PPIs identified by Gordon *et al.* (*Nature* 2020) have 332 SARS-CoV-2 specific PPIs, all PPIs were tested in VeroE6 and several key PPIs (including the ACE2-spike protein) were lost in this dataset. We therefore collected differentially expressed genes (primary bronchial epithelial cells) and proteins (human Caco-2 cells) in diverse SARS-CoV-2 infected cell lines to overcome the limitations of VeroE6. We agree with the Reviewer that PPI data and differentially expressed genes/proteins in different cell lines or tissues may contain false positives as well. We have acknowledged this limitation and have added more explanations on page 30 of the revised manuscript.

References for Comment #8

1. Maisonnasse P, Guedj J, Contreras V, Behillil S, Solas C, Marlin R, et al. Hydroxychloroquine use against SARS-CoV-2 infection in non-human primates. *Nature.* 2020. Epub 2020/07/23. doi: 10.1038/s41586-020-2558-4.

2. Hoffmann M, Mosbauer K, Hofmann-Winkler H, Kaul A, Kleine-Weber H, Kruger N, et al. Chloroquine does not inhibit infection of human lung cells with SARS-CoV-2. *Nature.* 2020. Epub 2020/07/23. doi: 10.1038/s41586-020-2575-3.

3. Gordon DE, Jang GM, Bouhaddou M, Xu J, Obernier K, White KM, et al. A SARS-CoV-2 protein interaction map reveals targets for drug repurposing. *Nature.* 2020. Epub 2020/05/01. doi: 10.1038/s41586-020-2286-9.

9. How did the authors perform functional enrichment analysis for SARS2-PPI and HCoV-PPI?

Response: We have added the detailed description (page 37) for **Functional enrichment analysis**: we used the online tool Enrichr (Kuleshov et al., *Nucleic Acids Res* 2016) and examined the enrichment of pathways from the Kyoto Encyclopedia of Genes and Genomes (KEGG) and Gene Ontology (GO) biological process.

Reference for Comment #9

Kuleshov MV, Jones MR, Rouillard AD, Fernandez NF, Duan Q, Wang Z, et al. Enrichr: a comprehensive gene set enrichment analysis web server 2016 update. *Nucleic Acids Res.* 2016;44(W1):W90-7. Epub 2016/05/05. doi: 10.1093/nar/gkw377.

10. The differential expressed genes identified from transcriptome profiles can be very different from proteomic data.

Response: We agree that differentially expressed genes identified from transcriptomic profiles can be very different from proteomic data. Due to the disease heterogeneities of COVID-19 (Gupta et al., *Nature Medicine* 2020), we posited that combining transcriptome profiles and proteomics data from diverse COVID-19 relevant cell lines or tissues may provide complementary, molecular information to overcome disease heterogeneities of COVID-19. We have added more explanations in the revised manuscript (page 30).

Reference for Comment #10

Gupta A, Madhavan MV, Sehgal K, Nair N, Mahajan S, Sehrawat TS, Bikdeli B, Ahluwalia N, Ausiello JC, Wan EY, Freedberg DE, Kirtane AJ, Parikh SA, Maurer MS, Nordvig AS, Accili D, Bathon JM, Mohan S, Bauer KA, Leon MB, Krumholz HM, Uriel N, Mehra MR, Elkind MSV, Stone GW, Schwartz A, Ho DD, Bilezikian JP and Landry DW. Extrapulmonary manifestations of COVID-19. *Nat Med*. 2020;26:1017-1032.

11. In Figs 2C, 2D, and 2G, what is the physical meaning of the ratio of nonsynonymous to synonymous substitutions (dN/dS) and evolutionary rate ratio?

Response: We calculated the nonsynonymous and synonymous substitution rate ratio (*dN/dS* ratio) (Hirsh et al., *Mol Biol Evol*. 2005) and the evolutionary rate ratio (Bezginov et al., *Mol Biol Evol*. 2013) as described in our previous study (Cheng et al., *Mol Biol Evol*. 2014). A *dN/dS* ratio below, equal to, or above 1 suggests purifying selection, neutral evolution, or positive Darwinian selection, respectively (Yang et al., *Trends Ecol Evol*. 2000). The evolutionary rate ratio was computed using the criterion that a ratio >1 indicates a fast rate and a ratio <1 indicates a slow rate (Bezginov et al., *Mol Biol Evol.* 2013). We have added these details in page 37 of the revised manuscript.

References for Comment #11

Hirsh AE, Fraser HB, Wall DP. Adjusting for selection on synonymous sites in estimates of evolutionary distance. *Mol Biol Evol.* 2005;22(1):174-7. Epub 2004/09/17. doi: 10.1093/molbev/msh265.

Bezginov A, Clark GW, Charlebois RL, Dar VU, Tillier ER. Coevolution reveals a network of human proteins originating with multicellularity. *Mol Biol Evol.* 2013;30(2):332-46. Epub 2012/09/15. doi: 10.1093/molbev/mss218.

Cheng F, Jia P, Wang Q, Lin CC, Li WH, Zhao Z. Studying tumorigenesis through network evolution and somatic mutational perturbations in the cancer interactome. *Mol Biol Evol.* 2014;31(8):2156-69. Epub 2014/06/02. doi: 10.1093/molbev/msu167.

Yang Z, Bielawski JP. Statistical methods for detecting molecular adaptation. *Trends Ecol Evol.* 2000;15(12):496-503. Epub 2000/12/15. doi: 10.1016/s0169-5347(00)01994-7.

12. In line 130 of page 7, the authors mentioned that "further compiled four additional virus-host gene/protein networks identified by different methods for comparisons". However, I am not sure how those virus-host gene/protein networks are identified, details regarding how to generate those networks and relevant references are missing.

Response: We have added a new Method section and **S3 Table** for these gene/protein sets.

"Collection of four additional virus-host gene/protein networks

To characterize the SARS-CoV-2 data sets, we downloaded four virus-host gene/protein networks for comparisons from previous studies: (1) 900 virus-host interactions identified by gene-trap insertional mutagenesis connecting 10 other viruses and 712 host genes [27]; (2) 2,855 virus-host interactions identified from RNA interference (RNAi) connecting 2,443 host genes and 55 pathogens [27]; (3) 579 host

proteins mediating translation of 70 innate immune-modulating viral open reading frames (viORFs) [34]; and (4) 1,292 host genes identified by co-immunoprecipitation and liquid chromatography-mass spectrometry (Co-IP+LC/MS) that mediate influenza-host interactions [35]. All details for those four virus-host gene/protein networks were provided in S3 Table."

References for Comment #12

[27] Cheng F, Murray JL, Zhao J, Sheng J, Zhao Z, Rubin DH. Systems Biology-Based Investigation of Cellular Antiviral Drug Targets Identified by Gene-Trap Insertional Mutagenesis. *PLoS Comput Biol.* 2016;12(9):e1005074. Epub 2016/09/16. doi: 10.1371/journal.pcbi.1005074.

[34] Pichlmair A, Kandasamy K, Alvisi G, Mulhern O, Sacco R, Habjan M, et al. Viral immune modulators perturb the human molecular network by common and unique strategies. *Nature.* 2012;487(7408):486-90. Epub 2012/07/20. doi: 10.1038/nature11289.

[35] Watanabe T, Kawakami E, Shoemaker JE, Lopes TJ, Matsuoka Y, Tomita Y, et al. Influenza virus-host interactome screen as a platform for antiviral drug development. *Cell Host Microbe.* 2014;16(6):795-805. Epub 2014/12/04. doi: 10.1016/j.chom.2014.11.002.

13. The network proximity measure was used to measure the distance of two genes/proteins in a protein-protein interaction network, however, the importance of the centrality of the nodes/proteins in the PPI network was ignored.

Response: We thank the Reviewer for this good point. We have calculated the eigenvector centrality of the genes in the asthma-COVID-19 and IBD-COVID-19 networks and added the results to **Fig. 5B** and **Fig. 6I**. We found that the highlighted genes (such as *IRAK3* and *ADRB2*) in the original manuscript have relatively high eigenvector centralities (top 5 and top 2, respectively) compared to other genes in the networks (**Fig. 5B** and **Fig. 6I**), which is consistent with our network proximity-based findings. We have added these new findings and more explanations in the revised manuscript (page 42).

14. The authors mentioned that "We next performed network-based drug repurposing using the existing knowledge of the drug-target network." and "Using our state-of-the-art network proximity framework, we measured the "closest" proximities of nearly 3,000

drugs". However, details about the drug-target network, the network proximity framework, and the nearly 3,000 drugs are missing.

Response: We have uploaded the network proximity results of the 3000 drugs to the GitHub page and provided as **S6 Table** as well. The details of the construction of the drug-target network and network proximity measure are added to the revised Methods section. The network proximity codes are available in this GitHub repository: https://github.com/ChengF-Lab/COVID-19_Map

15. The authors then computationally found 34 drugs that are associated with SARS-CoV-2 data sets, how did the authors rank those 3,000 drugs? how many of those 34 drugs are being tested, or have been tested in clinical trials and have positive effects for COVID-19 patients? The authors validated the efficacy of melatonin, one of those 34 drugs, on COVID-19 patients using their medical records. I am not sure if melatonin was ranked as the top-one among those 34 drugs? Not sure what are the differences between the drug-repositioning method proposed in this paper and in the authors' previous publication (ref 27), which used a similar network-based approach.

Response: We selected drug candidates using subject matter expertise based on a combination of factors: (i) strength of the network-based and bioinformatics-based predictions (a higher network proximity score [**S7 Table**] and significant GSEA score); (ii) literature-reported antiviral activities or ongoing clinical trial information; (iii) availability of sufficient patient data for meaningful evaluation (exclusion of infrequently used medications) from our COVID-19 registry database; and (iv) well-defined antiviral mechanisms-of-action (such as anti-inflammatory or immune modulators). Applying these criteria resulted in 34 drug candidates. Among 34 drug candidates, 16 drugs have reported antiviral effects and 8 drugs are in clinical trials for COVID-19 (5 clinical trial drugs in original submission). We have added all evidence for 34 drugs in **S7 Table** and add more explanations in the revised manuscript (page 21).

Among 34 drugs, melatonin is not the top-one candidate, but it is on top-ranked drug candidate. Several very top-ranked drugs (such as cancer drugs) are infrequently used medications (under-power) in our COVID-19 registry database, so they were excluded from our patient-based data validation analyses. We therefore selected two

top-ranked drugs, melatonin and carvedilol, which have enough number of patient data points to allow meaningful analysis of drug-disease outcome relationship with COVID-19 in our cohort. We have added more explanations in the revised manuscript.

16. I am not sure how cell types in Figs 5C, 5F, 6C, and 6D are annotated? Are there any control samples in the single-cell RNA-seq analysis?

Response: The lung and primary human bronchial epithelial cells were from normal tissues and have cell types annotated in the original study (Lukassen et al., *EMBO J.* 2020). The Crohn's disease cells were from both inflamed and uninflamed (as controls) regions from the ileal samples of 8 patients. Their cell types were annotated using the marker genes from the original paper (Martin et al., *Cell* 2019) and a recent meta-analysis paper using the same data set (Zhang et al., *Gut.* 2020). The expression of these marker genes is provided in **S20 and S21 Figs**. We have added these details in the revised manuscript (page 43).

References for Comment #16

1. Lukassen S, Chua RL, Trefzer T, Kahn NC, Schneider MA, Muley T, et al. SARS-CoV-2 receptor ACE2 and TMPRSS2 are primarily expressed in bronchial transient secretory cells. *EMBO J.* 2020;39(10):e105114. Epub 2020/04/05. doi: 10.15252/embj.20105114.

2. Martin JC, Chang C, Boschetti G, Ungaro R, Giri M, Grout JA, et al. Single-Cell Analysis of Crohn's Disease Lesions Identifies a Pathogenic Cellular Module Associated with Resistance to Anti-TNF Therapy. *Cell.* 2019;178(6):1493-508 e20. Epub 2019/09/03. doi: 10.1016/j.cell.2019.08.008.

3. Zhang H, Kang Z, Gong H, Xu D, Wang J, Li Z, et al. Digestive system is a potential route of COVID-19: an analysis of single-cell coexpression pattern of key proteins in viral entry process. *Gut.* 2020;69(6):1010-8. doi: 10.1136/gutjnl-2020-320953.

17. In Fig7.B there are four different bars with different colors, what does the PS-matched model 1 and PS-matched model 2 mean? There are two different OR model1s indicated by different colors, however, are these OR model1s independent? Besides, are four different models (variables) in Fig7.B independent?

Response: We have revised the original Fig. 7B as the new **Fig. 8**. We added more explanations in the revised **Fig. 8** legend and the revised manuscript for four different models we used. To be specific, we revised the Method section as follows (page 47):

“*Propensity score (PS) was used to match patients to reduce various confounding factors. Four models, from less to more stringent in terms of patient matching and odds ratios (OR) adjustment, were performed: (1) model 1 was matched using age, gender, race, and smoking without adjustment for the odds ratio; (2) model 2 was matched using age, gender, race, and smoking, and the odds ratio of COVID-19 was adjusted by age, gender, race, and smoking; (3) model 3 was matched using age, gender, race, smoking, coronary artery disease, diabetes, hypertension, and COPD without adjustment for the odds ratio; and (4) model 4 was matched using age, gender, race, smoking, coronary artery disease, diabetes, hypertension, and COPD, and the odds ratio of COVID-19 was adjusted by age, gender, race, smoking, coronary artery disease, diabetes, hypertension, and COPD.*”

Responses to Reviewer #2

This paper addresses the network interpretation of higher risk of morbidity and mortality of COVID-19 patients with one or more other common diseases utilizing integrative network analysis of transcriptomics, proteomics, and human interactome. Utilizing bulk and single cell RNA-seq data together with differential metabolite information (only for asthma patients), the authors provided insights on shared pathobiology of COVID-19 patients with asthma and inflammatory bowel diseases. The authors of this paper utilized their earlier developed in-silico drug repurposing approaches on COVID-19 clinical registry database and prioritized existing FDA-approved drugs as potential therapeutic candidates. Overall authors utilized all possible data sources and network-inference state of the art methods in their integrative analysis. The question remains however, with regard to whether these methods are good enough to yield substantial predictions. Below are the major concerns that need to be carefully further addressed before this paper can be considered for publication.

Response: We thank the Reviewer for the great summary and overall positive comments. We have intensified the rigor of our computational analysis and performed new extensive experiments to specifically address these critiques.

1. One intrinsic limitation of the authors' method is that directionalities are in general not being taken into account in the various networks they built and/or used. For example, it seems that whether a viral protein activates or inhibits a host protein, or whether a gene is upregulated or downregulated in a disease is not being considered, and this can make the interpretation of results difficult or give rise to ambiguities. As an example of this issue, although the authors have identified proximity between the SARS-CoV-2 network and the asthma network with several shared nodes (Fig. 5A), when comparing the differential expression (DE) profiles in asthma to that in SARS-CoV-2 infection (Fig. 5B), there does not seem to be significant concordance in terms of the direction of DE. Notably, IL6 increased in SARS-CoV-2 infection but decreased in asthma. Will the same findings still hold if the directionality is taken into account properly? We think that this is an important issue that should be addressed appropriately.

Response: We agree with the Reviewer that it is important to take into account the directionalities during human interactome network analysis. However, to date, there are no comprehensive SARS-CoV-2 virus-host PPIs that have directionalities, such as a viral protein activates or inhibits a host protein in a systematic way. For the human interactome, we don't have a systematic human protein-protein interactome with well-documented directionalities for each protein-protein interaction as well. In addition, a previous study has shown that integration of the directionality of the human interactome didn't change the results of network proximity measure (Menche et al., *Science* 2015).

To respond the Reviewer's concerns, we have re-computed the network proximity of up- or down- regulated genes in SARS2-DEG and differentially expressed genes (DEGs) from the two asthma data sets. A total of four combinations was performed for each asthma data set: asthma-DEG-up + SARS-DEG-up, asthma-DEG-up + SARS-DEG-down, asthma-DEG-down + SARS-DEG-up, and asthma-DEG-down + SARS-DEG-down. We found more significant network proximity (lower z-score and p-value) when we incorporated the directionalities of the DEGs. We have added these new findings and more explanations in the revised manuscript (page 33) (**S17 Fig**, **Extended Figure 2**).

Z score		SARS2-DEG Up DEG	SARS2-DEG Down DEG
GSE63142	Up DEG	-1.3	-0.2
	Down DEG	-1.5	-1.7
GSE130499	Up DEG	-1.2	0.2
	Down DEG	-2.8	-1.7

P value		SARS2-DEG Up DEG	SARS2-DEG Down DEG
GSE63142	Up DEG	0.08	0.38
	Down DEG	0.05	* 0.04
GSE130499	Up DEG	0.07	0.52
	Down DEG	* 0.01	* 0.04

-2 Z score 2

* P < 0.05

Extended Figure 2. Network proximity analysis of asthma and COVID-19 taking into consideration the directionalities of the differential gene expression. The up- and down-expressed genes in the two asthma data sets (GSE63142 and GSE130499, severe vs. control) were computed against the up- and down- expressed genes from the SARS2-DEG data set. Overall, the results show more significant network proximities and smaller z scores than when the direction is not considered as in Fig 4.

Reference for Comment #1

Menche J, Sharma A, Kitsak M, Ghiassian SD, Vidal M, Loscalzo J, et al. Disease networks. Uncovering disease-disease relationships through the incomplete interactome. *Science*. 2015;347(6224):1257601. Epub 2015/02/24. doi: 10.1126/science.1257601.

2. The different coronavirus datasets were from different cell types, for example the SARS2-DEG was based on data in primary bronchial epithelial cells, while the SARS2-DEP was based on Caco-2 colorectal cancer cell line, and the SARS2-PPI was based on HEK273T cell line. Further, the genes associated with each disease should all act in a context-specific manner in the corresponding tissue types where each of the diseases manifest. It's questionable whether the network within one context can still largely hold if transferred to a different context (e.g. tissue type). The authors have implicitly studied the related issue of cell/tissue type-specific expression. If the tissue type-specific expression information is explicitly used to refine the various SARS2 network separately and specifically for each tissue type, will the findings on proximity with disease networks still hold? This potentially serious confounding factor cannot be ignored and needs to be carefully addressed.

Response: We utilized SARS-CoV-2 virus-host PPIs and differentially expressed genes and proteins derived from different cells or tissues as single cell line has limitations for COVID-19 drug functional test. For example, two recent studies suggested that chloroquine or hydroxychloroquine showed ideal antiviral activities in African green monkey kidney cells but not in *a model of reconstituted human airway epithelium or TMPRSS2-positive lung cell line Calu-3* (Maisonnasse et al., *Nature* 2020; Hoffmann et al., *Nature* 2020). These studies showed that cell lines mimicking important aspects of respiratory epithelial cells should be used when analyzing the antiviral activity of drugs targeting host cell functions. In this study, we posited that SARS-CoV-2 virus-host PPIs collected from different COVID-19 relevant cell lines or tissues may provide complementary information for better understanding of pathobiology of COVID-19.

We agreed with the Reviewer that the genes associated with each disease should all act in a context-specific manner in the corresponding tissue types where each of the diseases manifest. However, recent studies have suggested that COVID-19 is a systematic disease which has impacts on multiple human tissues and organs (Gupta et al., *Nature Medicine* 2020). We therefore cannot use a single cell type or tissue/organ to explore heterogeneities of COVID-19.

To respond the Reviewer's concerns, we inspected tissue-specificity of diseases using available RNA-sequencing data (v8) across 33 tissue types from GTEx database (The GTEx Consortium, *Nat Genet.* 2013). When we recomputed the network proximity as in **Fig. 4A** using only genes that have positive tissue specificities in the associated disease, we noticed overall consistent results with some noticeable differences (**S15 Fig**, **Extended Figure 3**). We have added these new findings and more explanations in the revised manuscript (pages 32 and 33).

References for Comment #2

Maisonnasse P, Guedj J, Contreras V, Behillil S, Solas C, Marlin R, et al. Hydroxychloroquine use against SARS-CoV-2 infection in non-human primates. *Nature.* 2020. Epub 2020/07/23. doi: 10.1038/s41586-020-2558-4.

Hoffmann M, Mosbauer K, Hofmann-Winkler H, Kaul A, Kleine-Weber H, Kruger N, et al. Chloroquine does not inhibit infection of human lung cells with SARS-CoV-2. *Nature.* 2020. Epub 2020/07/23. doi: 10.1038/s41586-020-2575-3.

Gupta A, Madhavan MV, Sehgal K, Nair N, Mahajan S, Sehrawat TS, et al. Extrapulmonary manifestations of COVID-19. *Nat Med.* 2020;26(7):1017-32. Epub 2020/07/12. doi: 10.1038/s41591-020-0968-3.

The GTEx Consortium. The Genotype-Tissue Expression (GTEx) project. *Nat Genet.* 2013;45(6):580-5. Epub 2013/05/30. doi: 10.1038/ng.2653.

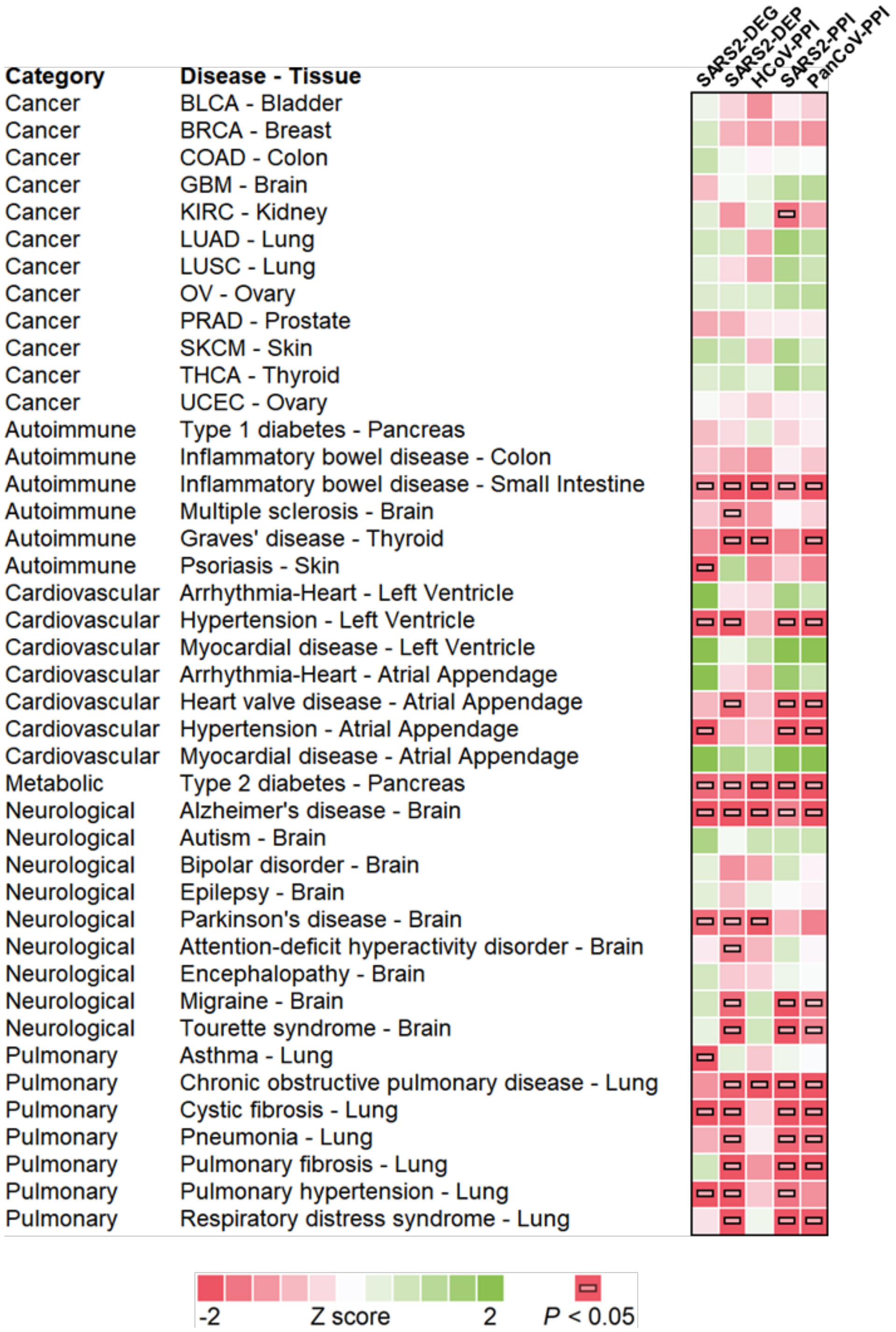

Extended Figure 3. Disease manifestations associated with COVID-19 quantified by network proximity measure using tissue-specific genes for each disease. The disease-associated genes were filtered by their tissue specificity. Tissues considered are shown after the disease names. Only genes with positive specificity were retained for the network analysis. After filtering, diseases with less than 5 genes were removed from the evaluation.

3. In the 'Validating drug-outcome relationships on COVID-19 using patient data' under Results section, authors utilized their earlier developed approaches of network proximity, GSEA analysis and PS-score matching methods to prioritize the drug candidates. The basis for selection of the final drug melatonin seems weak. The authors didn't mention anywhere how many drugs they finally considered in their analysis and with what frequency each of them was used by the patients.

Response: We selected final drug candidates for patient database validation using subject matter expertise based on a combination of factors: (i) strength of the network-based and bioinformatics-based predictions (a higher network proximity score and significant GSEA score); (ii) literature-reported antiviral activities or ongoing clinical trial information; (iii) availability of sufficient patient data for meaningful evaluation (exclusion of infrequently used medications) from our COVID-19 registry database; and (iv) well-defined antiviral mechanisms-of-action (such as anti-inflammatory or immune modulators. Applying these criteria resulted in 34 drug candidates from over 3,000 screened drugs. Among 34 drug candidates, we selected two drugs, melatonin and carvedilol, have enough patients (**Table 1**) for a meaningful analysis of the odds ratio of COVID-19 in our cohort. We have discussed the limitation of patient database validation on commonly used medications only in the revised Discussion section (page 31).

4. It is not clear whether the drugs (including melatonin) are being taken by the patients before or at the time of being tested positive, if before then what is the time interval between the drug consumption and testing, for how long have the patient been taking the drug, and how such information are being used in terms of selecting the samples to include in the analysis, as well as in the analytical model during the analysis. Such details are critical for the interpretation of the results, and an overview should be provided in the main text (Results or Methods) with comprehensive additional details in the Supplementary Materials.

Response: We collected medication information that patients were actively taking at the time of testing via the REDCap tool. We have added these details in the revised Methods section (page 46).

In this study, we utilized two types of drug-outcome observational studies: 1) melatonin users versus the same number of non-melatonin users matched by propensity score; and 2) active comparator design: melatonin users versus the same number of comparator users matched by propensity score as well. We used angiotensin II receptor blockers (ARBs) or angiotensin-converting enzyme inhibitors (ACEIs) as two comparators as both ARBs and ACEIs were not associated with risk of SARS-CoV-2 infection in several recent studies (Vaduganathan et al., *N Engl J Med 2020*; Jarcho et al., *N Engl J Med 2020*; Mehta et al., *JAMA Cardiology 2020*). To be specific, we used propensity score to adjust for age, sex, race, smoking history, and various disease comorbidities (coronary artery disease, diabetes, hypertension, and COPD) during all drug-outcome analysis, including active comparator design. We agreed with Reviewer that other drug information (such as the time interval between the drug consumption and testing, and dose) are important factors as well. We have discussed these limitations and more explanations in the revised manuscript (pages 24, 25, 29, and 31).

References for Comment #4

1. Vaduganathan M, Vardeny O, Michel T, McMurray JJV, Pfeffer MA, Solomon SD. Renin-Angiotensin-Aldosterone System Inhibitors in Patients with Covid-19. *N Engl J Med.* 2020;382(17):1653-9. Epub 2020/04/01. doi: 10.1056/NEJMsr2005760.

2. Jarcho JA, Ingelfinger JR, Hamel MB, D'Agostino RB, Sr., Harrington DP. Inhibitors of the Renin-Angiotensin-Aldosterone System and Covid-19. *N Engl J Med.* 2020;382(25):2462-4. Epub 2020/05/02. doi: 10.1056/NEJMe2012924.

3. Mehta N, Kalra A, Nowacki AS, Anjewierden S, Han Z, Bhat P, et al. Association of Use of Angiotensin-Converting Enzyme Inhibitors and Angiotensin II Receptor Blockers With Testing Positive for Coronavirus Disease 2019 (COVID-19). *JAMA Cardiol.* 2020. Epub 2020/05/06. doi: 10.1001/jamacardio.2020.1855.

5. Whatever drug list authors provided in Figure 7A, it is very obvious that all drugs were not used by all the patients in similar frequency. Melatonin is a very common drug compared to other drugs listed in that figure. So all these statistical tests are not at all applicable for all these drugs. In other words, this validation is not very useful in the COVID-19 patients' context, where intake of medicines is not homogeneous among patients.

Response: We agreed with the Reviewer that pharmacoepidemiologic validation is limited for commonly used drugs, like melatonin and carvedilol we studied. However, this is a general limitation for all pharmacoepidemiologic validation for all human diseases, not COVID-19 specific limitation. Based on the current growth curve of our COVID-19 patient database at the Ohio and Florinda hospitals, we may be able to test more drugs in the near future by significant increase of patients from our COVID-19 registry database and our integrative team are actively working on it. We have added more explanations for this general limitation in the revised manuscript (page 31).

6. Can the authors prioritize drugs in a more context-specific manner rather than one drug for all? Likewise, can they prioritize drugs in a more similar group of patients, like for asthma patients or for IBD patients or for hypertension patients?
Response: We thank the Reviewer for this great point. We have performed new subgroup analysis for melatonin and carvedilol in asthma, diabetes, and hypertension patients. We didn't inspect IBD patients as it has low statistical power by small number of IBD patients in our current COVID-19 registry database. The results show that different subgroups react differently to melatonin or carvedilol intakes in terms of the likelihood of a positive laboratory test result for SARS-CoV-2 (**S12 Fig**, **Extended Figure 4**). For example, the protective effect of melatonin was more significant in diabetes patients (OR = 0.52, 95% CI 0.36-0.75) than in asthma patients (OR = 0.61, 95% CI 0.36-1.06) or hypertension patients (OR = 0.80, 95% CI 0.61-1.05). In addition, we also checked efficiency of melatonin and carvedilol for black Americans and white Americans separately (**Fig. 8C and 8D**, **S13 Fig**, **Extended Figure 5**). We found that the protective effect of melatonin was more significant in the black Americans (OR = 0.48, 95% CI 0.31-0.75) than in the white Americans (OR = 0.77, 95% CI 0.57-1.04). We have added these new results and more explanations in the revised Results section (pages 25 and 26).

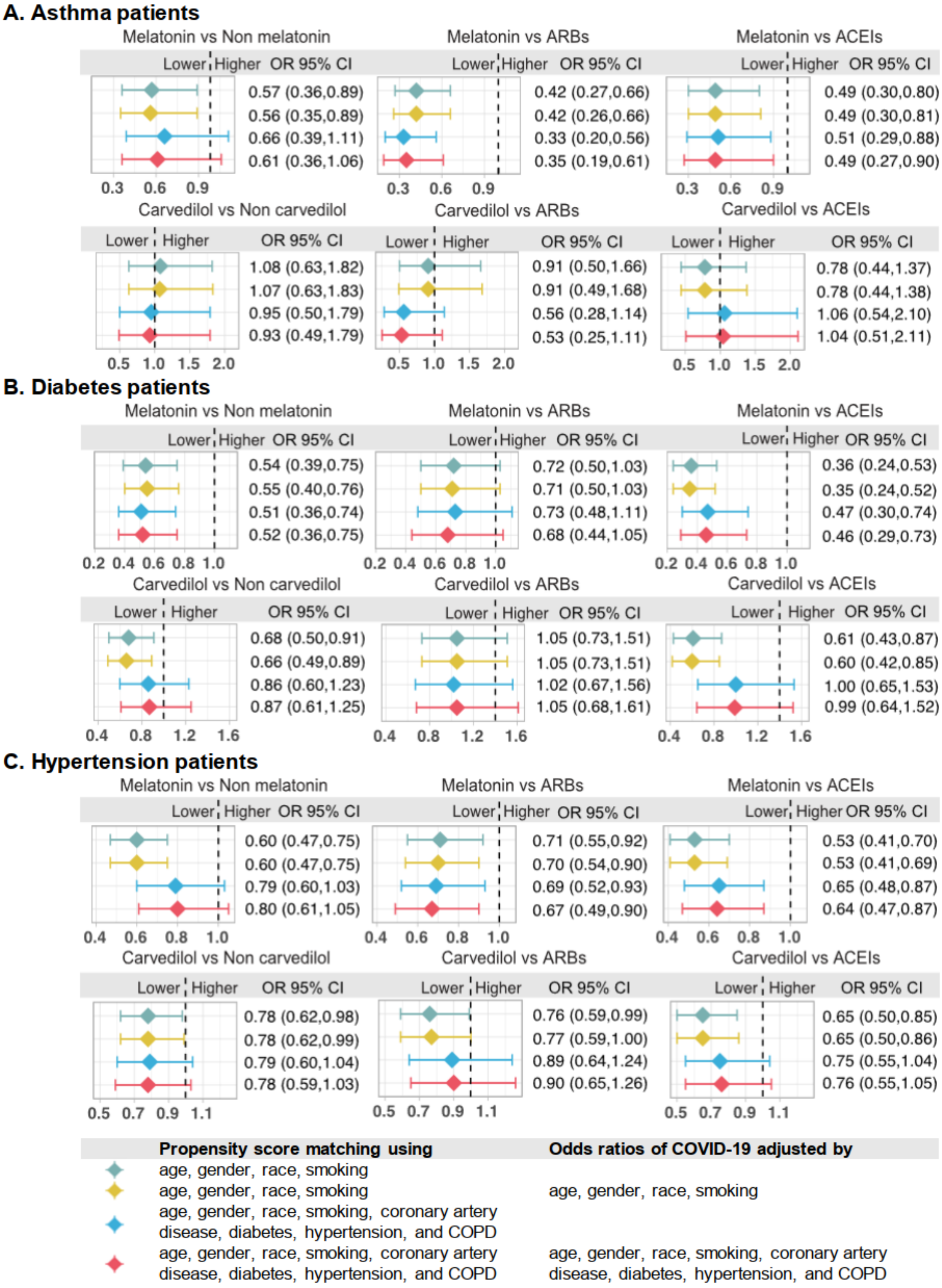

Extended Figure 4. Patient-based validation of drug repurposing for COVID-19 using three different subgroups, (A) asthma, (B) diabetes, and (C) hypertension. Four models were evaluated. These models were matched and adjusted using different variables as shown in the table. The variable that was used to extract each patient subgroup was not used for propensity score matching or odds ratios adjustment. ACEIs, angiotensin-converting enzyme inhibitors. ARBs, angiotensin II receptor blockers.

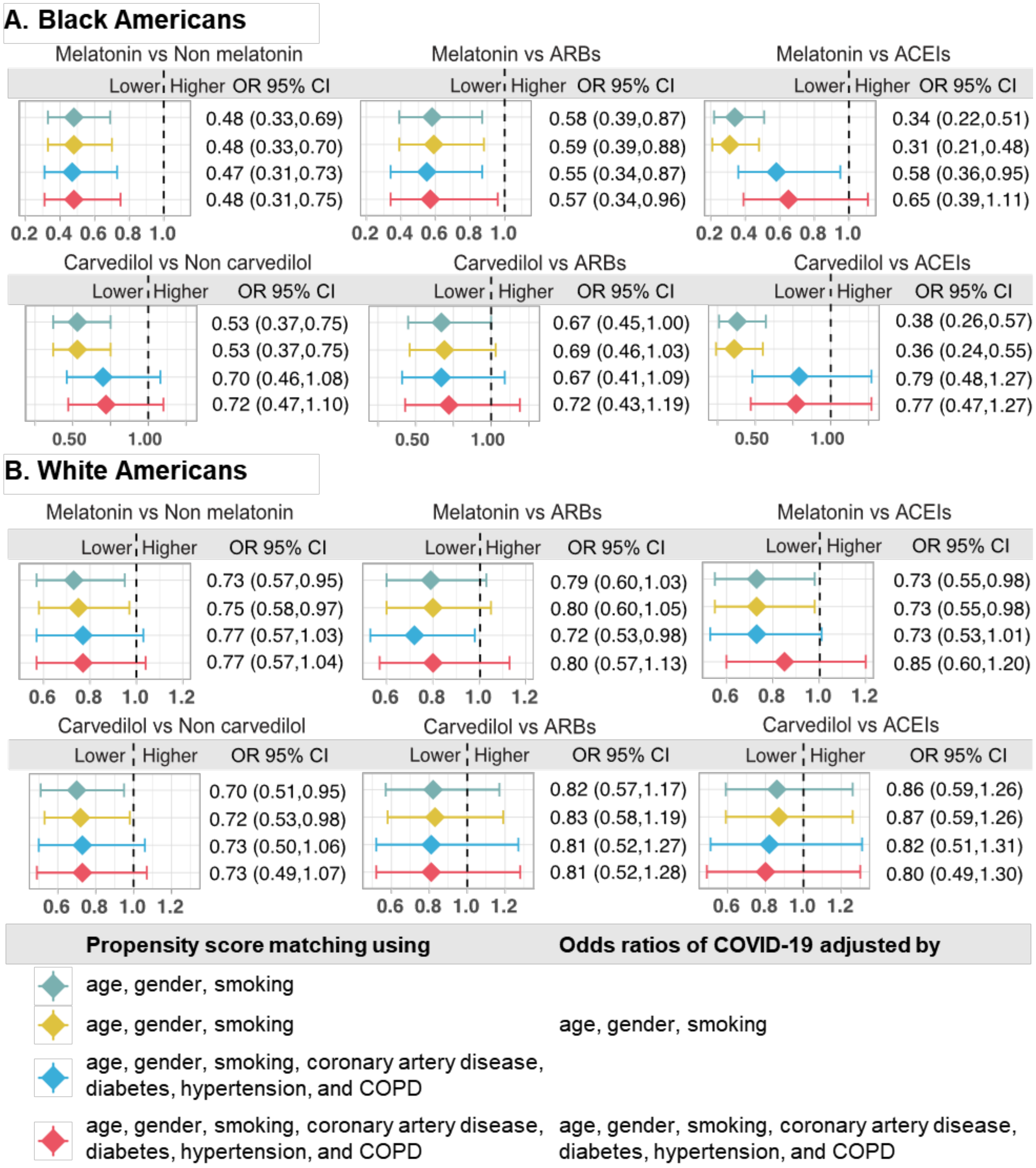

Extended Figure 5. Comparison of the patient validation results of melatonin and carvedilol usages in black Americans and white Americans respectively.

7. In many places, the authors just provided some numbers without any biological implication. There is no explanation of such variability in numbers or what are the biological consequences of those. For example, in Figure 2, the authors presented the

data for PanCoV-PPI which is a combination of HCoV-PPI and SARS2-PPI. It is well known that SARS2-PPI and HCoV-PPI networks are not very similar. In that case, all these numbers are not very useful towards the overall theme of the paper.

Response: We thank the Reviewer for this critique. We agreed with the Reviewer that SARS2-PPI and HCoV-PPI networks are not very similar. We combined SARS2-PPI and HCoV-PPI networks as we found several significant limitations of SARS2-PPIs. For example, SARS2-PPIs are identified from African green monkey kidney cells (VeroE6). Two recent studies suggested that chloroquine or hydroxychloroquine showed ideal antiviral activities in African green monkey kidney cells (VeroE6) but not in a model of reconstituted human airway epithelium or TMPRSS2-positive lung cell line Calu-3 (Maisonnasse et al., *Nature* 2020; Hoffmann et al., *Nature* 2020). In addition, multiple well-known PPIs (including ACE2-spike protein) are lost in SARS2-PPIs but are included in HCoV-PPI.

We used HCoV-PPI as pan-coronavirus PPIs as we pursue to identify broad-spectrum antiviral medications for SARS-CoV-2 and other emerging coronavirus if broadly applied of our network medicine framework. We have added more rationale and explanation why we combined SARS2-PPI and HCoV-PPI networks in the revised manuscript (pages 30 and 31).

We apologized that we did not provide the detailed explanations for each number in our original manuscript as our manuscript was very long. Now, we have added more explanations for each number in the revised manuscript based on our sizeable efforts, including 8 main Figures, 21 Supplementary Figures, and 7 Supplementary Tables, in total 72 pages in main manuscript.

References for Comment #7

1. Maisonnasse P, Guedj J, Contreras V, Behillil S, Solas C, Marlin R, et al. Hydroxychloroquine use against SARS-CoV-2 infection in non-human primates. *Nature.* 2020. Epub 2020/07/23. doi: 10.1038/s41586-020-2558-4.

2. Hoffmann M, Mosbauer K, Hofmann-Winkler H, Kaul A, Kleine-Weber H, Kruger N, et al. Chloroquine does not inhibit infection of human lung cells with SARS-CoV-2. *Nature.* 2020. Epub 2020/07/23. doi: 10.1038/s41586-020-2575-3.

8. The authors utilized the network proximity measure to evaluate the connectivity and closeness of other diseases with COVID-19. It is now well known a variety of underlying health conditions are risk factors for covid-19 patients, including children with rare diseases, like cerebral palsy or mental conditions. In such scenarios, network proximity is not a very useful measure for identifying this high morbidity and mortality risk. What is the authors take on that?
Response: We agreed with the Reviewer that our network proximity analysis only can focus on the diseases having well-known genetic information. For children with rare diseases, like cerebral palsy or mental conditions, we cannot perform network proximity analysis as we lack well-known genes/proteins associated with these conditions. We have added discussion about this limitation in the revised manuscript (page 31).

9. We feel that many parts of the writing are inaccurate or confusing and can be improved (examples given below). We recommend the authors to further refine the writing so that it is easier for the readers to understand:
(a) Line 214, the authors write "these diseases can be targeted directly or interact with the 215 targets of SARS-CoV-2 or other HCoVs." We think it is actually meant that "the disease genes can interact with the viral proteins either directly or indirectly via another host protein".
Response: We changed this sentence as suggested: "*Shown in Fig. 3A, the disease genes can interact with the SARS-CoV-2 viral proteins either directly or indirectly in the human protein-protein interactome.*" (page 12)

(b) Line 228, authors can explicitly mention which 8 comorbidities they are referring to here.
Response: We changed this part to "*We found that subjects with several disease comorbidities or risk factors have significantly higher risks in severe COVID-19 patients (Fig. 3B), including COPD, cardiovascular disease, stroke, diabetes mellitus, chronic kidney disease, hypertension, cancer, and history of smoking.*" (pages 12 and 13).

(c) Line 293, the term "endophenotype", which has a strict definition, may not be appropriate here, the authors may intend to write "molecular profile" or perhaps "molecular program".

Response: We have replaced "endophenotype" with "molecular profile" in the entire manuscript.

(d) Line 297, in multi-modal analysis, there is no clear explanation what authors exactly did here? There is no clear methodology for their multi-modal analysis in the Methods section.

Response: We added the following explanation in the revised manuscript (page 16) "*To be specific, we identified the enzymes in the network that are associated with altered metabolites in COVID-19 patients. Comparing the DEGs from asthma patients and DEGs from COVID-19 patients, we aim to find the common genes/proteins or interacting proteins in these patient groups. Using network analyses (degree enrichment and eigenvector centrality), we can rank the importance of these genes. Last, we examined their expression in the cell types that are more susceptible to SARS-CoV-2 (expressing more ACE2 and TMPRSS2).*"

(e) Line 308, "matching the enzymes of the differential metabolites and the proteins in the PPI network", it's not clear whether enzymes for the synthesis, or degradation, or any transformation, or transportation, etc. of the metabolites were considered, and it seems that this is not explained elsewhere either.

Response: We have added a new method section to address the question (pages 42 and 43) "***Building the metabolite-enzyme network:*** *We built a comprehensive metabolite-enzyme network by assembling data from four commonly used metabolism databases: KEGG [137], Recon3D [138], the Human Metabolic Atlas (HMA) [139], and the Human Metabolome Database (HMDB) [140]. The metabolite-enzyme network contains 60,822 records of 6,725 reactions among 3,808 metabolites and 3,446 genes. Four types of enzyme functions were included in the network: biosynthesis, degradation, transformation, and transportation.*"

References for Comment #e

[137] Kanehisa M, Sato Y, Kawashima M, Furumichi M, Tanabe M. KEGG as a reference resource for gene and protein annotation. *Nucleic Acids Res.* 2016;44(D1):D457-62. Epub 2015/10/18. doi: 10.1093/nar/gkv1070.

[138] Brunk E, Sahoo S, Zielinski DC, Altunkaya A, Drager A, Mih N, et al. Recon3D enables a three-dimensional view of gene variation in human metabolism. *Nat Biotechnol.* 2018;36(3):272-81. Epub 2018/02/20. doi: 10.1038/nbt.4072.

[139] Pornputtapong N, Nookaew I, Nielsen J. Human metabolic atlas: an online resource for human metabolism. *Database (Oxford).* 2015;2015:bav068. Epub 2015/07/26. doi: 10.1093/database/bav068.

[140] Wishart DS, Feunang YD, Marcu A, Guo AC, Liang K, Vazquez-Fresno R, et al. HMDB 4.0: the human metabolome database for 2018. *Nucleic Acids Res.* 2018;46(D1):D608-D17. Epub 2017/11/16. doi: 10.1093/nar/gkx1089.

(f) Line 318 "Utilizing two bulk RNA-Seq data sets from asthma patients and healthy controls, we identified elevated expression of IRAK3 and ADRB2 in SARS-CoV-2 infected human bronchial epithelial cells." -- this is confusing.

Response: We changed this part to "*Utilizing two bulk RNA-Seq data sets (GSE63142 and GSE130499) of asthma patients compared to healthy controls, we identified that IRAK3 and ADRB2 had significantly elevated expression (FDR < 0.05) in asthma patients. Both genes also have significantly elevated expression SARS-CoV-2 infected human bronchial epithelial cells (Fig. 5B).*" (page 17)

(g) Line 428 "Validating drug-outcome relationships on COVID-19 using patient data", it seems to mean "evidence from the COVID-19 registry data that supports the predicted drug repurposing strategies".

Response: We have changed this section header to "*Evidence from the COVID-19 registry data that supports the predicted drug repurposing strategies*" as suggested.

Responses to Reviewer #3

The manuscript by Zhou and colleagues is submitted for consideration for publication to PLOS Biology. In this manuscript the authors tried to investigate pathogenesis, clinical manifestations and therapies COVID-19 using network medicine approach on clinical

and multi-omics data. The reason for this study is to understand molecular mechanisms of SARS-CoV-2 infection, to compare with other not-infectious diseases and to identify FDA-approved drugs as potential COVID-19 drug candidates through network-medicine findings and clinical data from a large COVID-19 clinical registry database. **The paper is well structured and exhaustive in every part, while the integrated approach, the authors have used, is original and very interesting.** Importantly, this theoretical finding might have practical significance via guiding both pharmaceutical and diagnostic research with the prospect to identify potential new biological targets. It can be recommended for publication upon addressing several concerns into some not clear parts.

Response: We thank the Reviewer for the support.

1. In introduction they report numbers of pandemics, but it needs to insert one or more references (e.g. John Hopkins University), while at row 54 they should add other references about network based-approach model based on comparative PPI interactomes with other HCoV and concept of Disease Map applied to COVID19.

Response: We have added the references and more explanations about concept of Disease Map applied to COVID19 in the revised manuscript.

References for Comment #1

1. Dong E, Du H, Gardner L. An interactive web-based dashboard to track COVID-19 in real time. *Lancet Infect Dis.* 2020;20(5):533-4. Epub 2020/02/23. doi: 10.1016/S1473-3099(20)30120-1.

2. Pfefferle S, Schopf J, Kogl M, Friedel CC, Muller MA, Carbajo-Lozoya J, et al. The SARS-coronavirus-host interactome: identification of cyclophilins as target for pan-coronavirus inhibitors. *PLoS Pathog.* 2011;7(10):e1002331. Epub 2011/11/03. doi: 10.1371/journal.ppat.1002331.

3. Fung TS, Liu DX. Human Coronavirus: Host-Pathogen Interaction. *Annu Rev Microbiol.* 2019;73:529-57. Epub 2019/06/22. doi: 10.1146/annurev-micro-020518-115759.

2. in Results they talk about S2 Table, containing "additional virus-host gene / protein networks identified by different methods for comparisons", but it is not clear how they selected these genes, where these data is from and what purpose it would serve.

Response: We have added references for the sources of these datasets in the revised Method section (page 35) with the revised Supplemental **S3 Table** (originally S2 table). They were used as comparisons to find common and unique network and bioinformatic characteristics of SARS-CoV-2. We also added the following sentence "*These virus-host gene/protein networks have been well-characterized and offer high quality data sets for comparisons.*" (page 8)

3. at page 19 they wrote: "These observations reveal common network relationship between COVID-19 and human diseases". In my opinion, this phrase could result obvious, due to wide previous literature about COVID19 produced up this moment and the pathogenic and molecular similarity with SARS-CoV.
Response: We have added the following to the limitation (page 31) "*Potential literature bias of disease-associated genes and the human interactome may also influence our findings. For example, well-studied genes in both COVID-19 and other diseases may explain the similarity of COVID-19 with other diseases, while the under-studied genes associated with both diseases may not be uncovered.*"

4. at page 20 they talk about the network-based relationships of the 64 diseases across the 6 categories to COVID-19, shown in Fig 4A, taking into account the proximity, Z scores and P values, as significance test. However this part results hard to understand: firstly, they should report the absolute numbers of how many genes they used for each Z-score test and p-value, because different sample size of genes provide the strengthness of associations.
Response: We analyzed the correlation between the z scores and number of genes for these five data sets and found that the number of genes is only weakly associated with z scores (maximum R^2=0.1468). We have added the following to the discussion (page 27) "*The number of genes associated with each disease varies. However, we did not notice any significant bias towards the network proximity z scores by different number of genes (S14 Fig).*" The results are added in **S14 Fig** and **Extended Figure 6**. The details of the number of genes and sources for each disease are also provided in **S4 Table**.

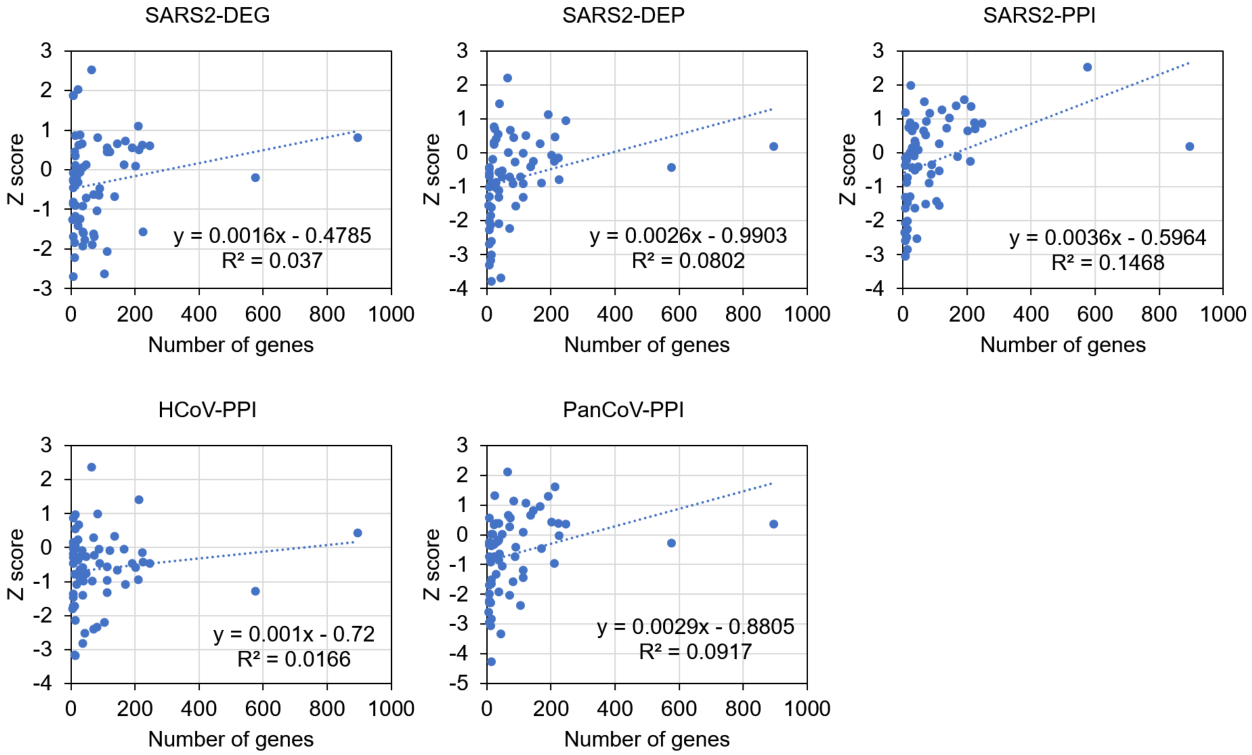

Extended Figure 6. Analysis of the effect of the number of genes associated with the diseases on the network proximity z scores. Each dot represents a disease (z score versus number of genes). No significant bias was observed for the number of genes. The maximum R^2 is 0.1468 from the SARS2-PPI data set.

5. Secondly, they must explain to biological function of genes tested and the pathway involved, because it is very difficult to figure out why they found strong significant network proximity with attention-deficit / hyperactivity disorder and not with other cardiovascular diseases, since vascular damage is one of most featured manifestations in severe COVID19 cases, or asthma.

Response: We thank the Reviewer for this great point. The nervous system and the immune system can communicate and affect each other by bidirectional interactions (Schiller et al., *Nature Reviews Immunology* 2020). Recent clinical studies have shown that SARS-CoV-2 infection was associated with multiple neurological syndromes (Paterson et al., *Brain* 2020; Cebrián et al., *Neurology* 2020; Gane et al., *Rhinology* 2020). To understand the association of SARS-CoV-2 infection and ADHD in the context of human protein interactome, we performed network connectivity analysis and found that many ADHD-associated genes are connected to SARS-CoV-2 host genes

(**Extended Figure 7**). Using SARS2-DEG, SARS2-DEP, SARS2-PPI, we found that PRKN and TAF1 appeared in all three subnetworks, and MTNR1A, EPHA5, GIT1, UBE2F, CTNNA3, and TUSC3 appeared in two subnetworks. For example, it has been suggested that the copy number variation of PRKN is associated with the genetic susceptibility of ADHD (Jarick et al., *Mol Psychiatry* 2014).

As for why we didn't see a strong network proximity association between COVID-19 and cardiovascular diseases, one potential explanation is that the vascular damage is caused by inflammatory adverse effects from SARS-CoV-2 infections. However, the cardiovascular disease-associated genes from HGMD are genetics driven findings. Another potential reason is tissue specificity of the gene expressions. For example, when we filtered the genes by their heart expression before network proximity analysis, heart valve disease become significant with SARS2-DEP, SARS2-PPI, and PanCoV-PPI (**S15 Fig**, **Extended Figure 3**).

References for Comment #5

1. Schiller M, Ben-Shaanan TL, Rolls A. Neuronal regulation of immunity: why, how and where? *Nat Rev Immunol.* 2020. Epub 2020/08/20. doi: 10.1038/s41577-020-0387-1.

2. Paterson RW, Brown RL, Benjamin L, Nortley R, Wiethoff S, Bharucha T, et al. The emerging spectrum of COVID-19 neurology: clinical, radiological and laboratory findings. *Brain.* 2020. Epub 2020/07/09. doi: 10.1093/brain/awaa240.

3. Cebrian J, Gonzalez-Martinez A, Garcia-Blanco MJ, Celdran-Vivancos D, Palacios EL, Reig-Rosello G, et al. Headache and impaired consciousness level associated with SARS-CoV-2 in CSF: A case report. *Neurology.* 2020;95(6):266-8. Epub 2020/07/10. doi: 10.1212/WNL.0000000000010213.

4. Gane SB, Kelly C, Hopkins C. Isolated sudden onset anosmia in COVID-19 infection. A novel syndrome? Rhinology. 2020;58(3):299-301. Epub 2020/04/03. doi: 10.4193/Rhin20.114.

5. Jarick I, Volckmar AL, Putter C, Pechlivanis S, Nguyen TT, Dauvermann MR, et al. Genome-wide analysis of rare copy number variations reveals PARK2 as a candidate gene for attention-deficit/hyperactivity disorder. *Mol Psychiatry.* 2014;19(1):115-21. Epub 2012/11/21. doi: 10.1038/mp.2012.161.

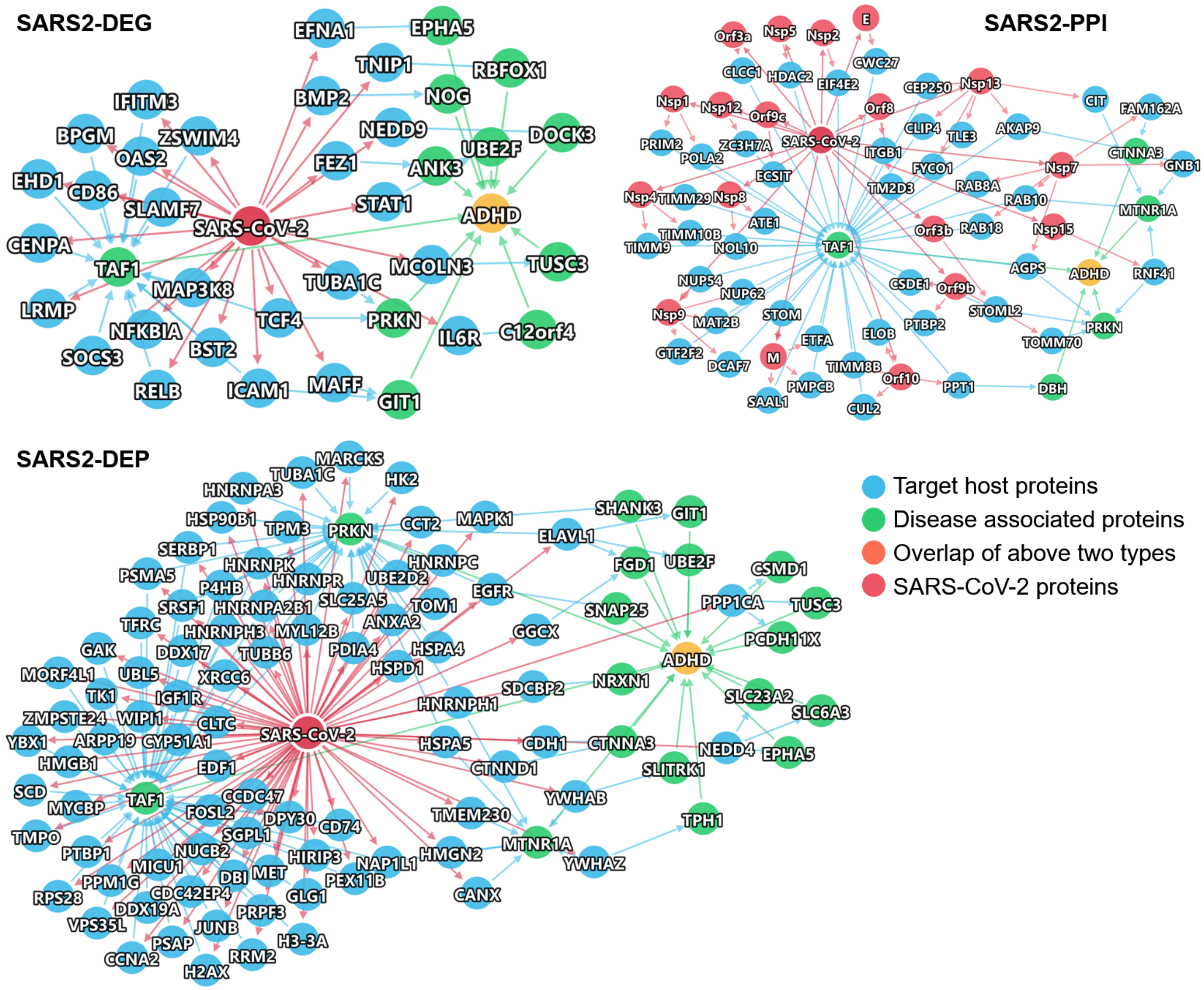

Extended Figure 7. Subnetworks between SARS-CoV-2 host genes/proteins with the disease-associated proteins of attention-deficit / hyperactivity disorder.

6. Moreover, for the networks in this figure, it is not clear why they chose sepsis and respiratory distress syndromes as example and it could result misleading.

Response: We chose sepsis and respiratory distress syndromes as two examples as these two complications are the main causes of mortality of SARS-CoV-2 infection in severe COVID-19 patients.

References for Comment #6

1. Fan E, Beitler JR, Brochard L, Calfee CS, Ferguson ND, Slutsky AS, et al. COVID-19-associated acute respiratory distress syndrome: is a different approach to management warranted? *The Lancet Respiratory Medicine.* 2020;8(8):816-21. doi: 10.1016/s2213-2600(20)30304-0.

2. Prescott HC, Girard TD. Recovery From Severe COVID-19: Leveraging the Lessons of Survival From Sepsis. *JAMA* 2020. Epub 2020/08/11. doi: 10.1001/jama.2020.14103.

7. at page 28 they analyzed data by a large-scale patient data from the Cleveland Clinic COVID-19 patient registry, evaluating the characteristics of melatonin and carvedilol. I noted that in SARS-CoV-2 positive patients there was a wide diversity in sample size between cases and controls (cases are ~ 2% of controls). I understand it was due to availability of COVID19 cases tested, but it should much more report in the limitation section.

Response: We have updated the patient validation results using our latest COVID-19 registry collected from March 8 to July 27, 2020. Currently there are **8,274** (31%) COVID-19 positive patients in the registry of 26,779 subjects, representing over four-fold increases in the COVID-19 positive patients compared to our original manuscript. We have added these new findings in the new **Fig. 8** and added more explanations in pages 25 and 26 of the revised manuscript.
