# Supplementary material for: A network medicine approach to investigation and population-based validation of disease manifestations and drug repurposing for COVID-19
Source: PLoS Biol. 2020 Nov 6;18(11):e3000970. doi: 10.1371/journal.pbio.3000970 (PMC7728249; doi:10.1371/journal.pbio.3000970)
Supplement: Attachment — Submitted filename: Response_to Editors comments.docx [file pbio.3000970.s042.docx]

# Manuscript #: PBIOLOGY-D-20-01653R2

# Responses to Editor:

a) Please address my Data Policy requests (see further down).

**Response:** We have made the following changes: we added S1-S11 Data. These files contain the underlying data for the figures. The original S7 Table was renamed to S6 Data. S1-S6 Tables are still provided since they contain more complete and raw data. We then added the sentence “The data underlying this figure can be found in S… Data” to each figure legend and added references to them in the manuscript for the first occurrence of each main figure.

b) Please clarify the funding information (it currently appears that you had no specific funding for this project, but this is unclear).

**Response:** We have added the below Funding information in the manuscript submission systems: “This work was supported by the National Institute of Aging (R01AG066707 and 3R01AG066707-01S1) and the National Heart, Lung, and Blood Institute (R00HL138272) to F.C. This work has been also supported in part by the VeloSano Pilot Program (Cleveland Clinic Taussig Cancer Institute) to F.C.”

c) Please go very carefully through the text and especially the Abstract, ensuring that you do *not* overstate your claims regarding melatonin or any other treatments herein. This is particularly important given the high-profile disinformation that has occurred during recent months. You must only claim what is supported by the evidence presented. The final clause ("and identifying melatonin for potential prevention and treatment of COVID-19.") seems excessively strong. Also, given that melatonin is an endogenous chemical, the mentions of melatonin (e.g. "melatonin is associated with a reduced likelihood....") presumably should read "melatonin usage" or similar, to avoid the impression that endogenous levels were measured.

**Response:** We have changed “melatonin” to “melatonin usage”. There are at least eight different ongoing clinical trials registered at clinicaltrials.gov to test the treatment effects of melatonin for COVID-19 patients: NCT04531748 (Our proposed Selective Estrogen Modulation and Melatonin in Early COVID-19 (SENTINEL) at the Cleveland Clinic); NCT04474483; NCT04409522; NCT04568863; NCT04530539; NCT04470297; NCT04353128; NCT04570254. After discussed with all co-authors, including infectious disease experts, we prefer to keep the original statement. To respond to the editor’s concerns, we have added more explanations in the revised Discussion.

d) Please update any assessment of the current state of COVID-19 treatments to ensure that any fast-moving developments are captured.

**Response:** We have added the latest status of the development for some COVID-19 treatments, including Remdesivir and Dexamethasone, in the revised Introduction section (page 6).

DATA POLICY:

You may be aware of the PLOS Data Policy, which requires that all data be made available without restriction: http://journals.plos.org/plosbiology/s/data-availability. For more information, please also see this editorial: http://dx.doi.org/10.1371/journal.pbio.1001797

Note that we do not require all raw data. Rather, we ask that all individual quantitative observations that underlie the data summarized in the figures and results of your paper be made available in one of the following forms:

1) Supplementary files (e.g., excel). Please ensure that all data files are uploaded as 'Supporting Information' and are invariably referred to (in the manuscript, figure legends, and the Description field when uploading your files) using the following format verbatim: S1 Data, S2 Data, etc. Multiple panels of a single or even several figures can be included as multiple sheets in one excel file that is saved using exactly the following convention: S1_Data.xlsx (using an underscore).

2) Deposition in a publicly available repository. Please also provide the accession code or a reviewer link so that we may view your data before publication.

Regardless of the method selected, please ensure that you provide the individual numerical values that underlie the summary data displayed in the following figure panels as they are essential for readers to assess your analysis and to reproduce it: All main and supplementary figure panels except Fig 1. NOTE: the numerical data provided should include all replicates AND the way in which the plotted mean and errors were derived (it should not present only the mean/average values).

IMPORTANT: I note that your supplementary files (Tables S1-S7) and Github deposition contain a significant amount of data; however their relationship to the specific Figure panels is unclear; please either supply the data that immediately underlies the Figures, or specify where it can be found; each main and supplementary Figure should have a clear citation to the underlying data (e.g. "The data underlying this Figure can be found in https://github.com/ChengF-Lab/COVID-19_Map"). It may be more appropriate to re-name Tables S2-S7 as Supplementary Data files.

Please also ensure that figure legends in your manuscript include information on where the underlying data can be found, and ensure your supplemental data file/s has a legend.

Please ensure that your Data Statement in the submission system accurately describes where your data can be found.

**Response:** We have made the following changes: we added S1-S11 Data. These files contain the underlying data for the figures. The original S7 Table was renamed to S6 Data. S1-S6 Tables are still provided since they contain more complete and raw data. We then added the sentence “The data underlying this Figure can be found in S… Data” to each figure legend and added references to them in the manuscript for the first occurrence of each main figure. We have double checked Data Statement in the submission system.

# Response to MANUSCRIPT REQUIREMENTS

1. Please complete the Blurb section of the submission form. This will be included in our weekly and monthly Electronic Table of Contents, sent out to users signed up to receive alerts from PLOS Biology. The blurb should be about 30-40 words long and is subject to editorial changes. It should, without exaggeration, entice people to read your manuscript. It should not be redundant with the title and should not contain acronyms or abbreviations. For examples, please see our Author Guidelines: http://journals.plos.org/plosbiology/s/revising-your-manuscript.

**Response:** We have added the below Blurb section in the manuscript submission systems: “*Using network medicine methodologies along with multiome (including SARS-CoV-2 and human interactome, transcriptome, and proteome) observations, Cheng and his colleagues identified widespread disease manifestations and drug repurposing candidates (including melatonin) for COVID-19 and validated their findings using large-scale patient registry database.*”

2. Please provide a detailed Financial Disclosure statement. This is published with the article, therefore should be completed in full sentences and contain the exact wording you wish to be published.

1. Please clarify all sources of funding (financial or material support) for your study. List the grants (with grant number) or organizations (with url) that supported your study, including funding received from your institution.

2. State the initials, alongside each funding source, of each author to receive each grant.

3. State what role the funders took in the study. If the funders had no role in your study, please state: “The funders had no role in study design, data collection and analysis, decision to publish, or preparation of the manuscript.”

4. If any authors received a salary from any of your funders, please state which authors and which funders.

If you did not receive any funding for this study, please state: “The authors received no specific funding for this work.”

**Response:** We have added the below Funding information in the manuscript submission systems: “This work was supported by the National Institute of Aging (R01AG066707 and 3R01AG066707-01S1) and the National Heart, Lung, and Blood Institute (R00HL138272) to F.C. This work has been also supported in part by the VeloSano Pilot Program (Cleveland Clinic Taussig Cancer Institute) to F.C.”

The funders had no role in study design, data collection and analysis, decision to publish, or preparation of the manuscript.

3. In your Data Availability Statement, please ensure you have provided all necessary access information in complete sentences. This statement will appear on the published version of your manuscript.

**Response:** Data Availability Statement: All data sets used in this study and their sources for downloading can be found in S1 Table. Source code, the human protein-protein interactome, and drug-target network can be downloaded from https://github.com/ChengF-Lab/COVID-19_Map. All other relevant data are within the paper and its Supporting Information files.

4. Please indicate the corresponding author on the title page with an asterisk (*) and include the corresponding author's email address. Only an asterisk and the email address itself are required. Please format as follows, omitting all other name, address and contact information:

* chengf@ccf.org

**Response:** We have modified this part as suggested.

5. Please ensure that all authors have at least one affiliation listed on the title page and that all listed affiliations are attributed to at least one author. Affiliations should be listed in ascending numerical order in the affiliation list and should match what is provided in the Authors Information in the submission form. Affiliations should include an institution, a city, and a country (no postcode). US affiliations need the state as well, written out in full (with no abbreviations).

**Response:** We have checked the affiliations, removed all postcodes, and changed abbreviations of the states to full name.

6. Please ensure that all author names are spelled correctly in the manuscript file and the submission form, including the use of a middle name/initials, and any special characters (accents, umlauts, etc.). Please place first/given names before last/family names. We noticed that Lara Jehi (Lara Lara) is spelled differently and ask that you ensure they match verbatim. Please be aware that correct spelling and formatting of author names in the article file is vital for accurate indexing in PubMed.

**Response:** We have used the right name (Lara Jehi) in manuscript submission system.

7. When referring to your Materials and Methods/Models/Methods section throughout the text, please use the full title rather than “Methods”, or change the title of the section to match the references in the text.

**Response:** We have added the name of the method sections when referring to them.

8. Please ensure that your Ethics Statement is available in the Methods section of your manuscript in its entirety, under the subheading “Ethics Statement”.

**Response:** We have added the Ethics Statement.

9. We have noted that you have the following present in your manuscript: Data Availability Statement. We will remove these sections from your manuscript file as this information is automatically pulled from the submission form. Please make sure that the information in the submission form is correct.

**Response:** We have removed Data Availability Statement in the main text and add it in the manuscript submission systems.

10. Please ensure that all Supporting Information files use the following format verbatim throughout the manuscript (including legends and all in-text citations): S1 Fig, S1 Table, S1 Text, S1 Data, etc. Each file type should start at 1 and increase numerically. Please note, however, that the file names themselves must use an underscore rather than a space, i.e. S1_Fig.tif, S1_Table.xlsx, S1_Text.doc, S1_Data.xlsx, S1_PRISMA_Checklist.docx, etc.

**Response:** We have checked the format for the supporting information files.

11. Please note that your submission cannot contain any references to generic "supporting information" files and instead must refer to specific files using our naming convention (S1 Fig, S1 Table, S1 Text, S1 Data, etc.)

**Response:** We have checked all references to the supporting information and used the naming convention as provided.

# Response to FIGURE REQUIREMENTS

1. Please ensure that all main figures are referenced in the manuscript as Fig 1, Fig 2, etc. (including capitalization), rather than Figure 1, Figure 2, etc. Please note, however, that the file names themselves must not include the space, i.e. Fig1.tif, Fig2.tif. Please ensure that all main figure files are clearly labeled as such in the File Inventory.

**Response:** We have prepared the figures according to the Figure Guidelines. They are referenced in the suggested format in the manuscript. All figures are in .tif (LZW compressed and flattened), have a DPI of 300, and do not exceed the maximum dimension and file size.

2. Please note that your figures do not currently comply with our publication requirements. For further instructions to assist with your Figure revisions, please refer to our Figure Guidelines at: http://journals.plos.org/plosbiology/s/figures.

If you need assistance, please contact us directly at graphics@jjeditorial.com so we can further help with this matter. Please send any relevant source files and tell us what application(s) and what operating system you are using.

- Some text in panel B of Figure 1 appears pixelated/low quality. Please remake this part of the figure using the original source, using the PLOS figure guidelines to ensure quality is preserved [http://journals.plos.org/plosbiology/s/figures].

- Figures 2, 3, 4, 5, 6, 7, and 8 are acceptable for publication.

**Response:** We have redone the text in Fig 1B to avoid it being pixelated. We also moved the figure captions such that they are placed immediately following the paragraph where they are first cited, according to the Figure Guidelines.

3. Please confirm that you either created the following figure panels, or that you will replace them with an open source alternative: illustrations in Fig. 1

Here are a few open source image websites you may wish to use:

- https://commons.wikimedia.org

- http://www.publicdomainpictures.net/

- https://openclipart.org/

**Response:** Thank you for the great resources. We created all the panels in Fig 1.
